# Decreasing incidence and mortality of lung cancer in Hungary between 2011 and 2021 revealed by robust estimates reconciling multiple data sources

**DOI:** 10.3389/pore.2024.1611754

**Published:** 2024-06-03

**Authors:** Gabriella Gálffy, Géza Tamás Szabó, Lilla Tamási, Veronika Müller, Judit Moldvay, Veronika Sárosi, Anna Kerpel-Fronius, Tamás Kardos, Edit Csada, Zsolt Pápai-Székely, Zoltán Szász, Zsolt Király, Gábor Hódi, Zsuzsanna Kovács, Éva Balogh, Krisztina Andrea Kovács, Miklós Darida, Viktória Buga, György Rokszin, Zsolt Abonyi-Tóth, Zoltán Kiss, Zoltán Vokó, Krisztina Bogos

**Affiliations:** ^1^ Department of Pulmonology, Pulmonology Center of the Reformed Church in Hungary, Törökbálint, Hungary; ^2^ MSD Pharma Hungary Ltd., Budapest, Hungary; ^3^ Department of Pulmonology, Semmelweis University, Budapest, Hungary; ^4^ Department of Pulmonology, National Korányi Institute of Pulmonology, Budapest, Hungary; ^5^ Department of Pulmonology, University of Szeged Albert Szent-Györgyi Medical School, Szeged, Hungary; ^6^ Faculty of Medicine, University of Pécs, Pécs, Hungary; ^7^ National Korányi Institute of Pulmonology, Department of Radiology, Budapest, Hungary; ^8^ Department of Pulmonology, University of Debrecen, Debrecen, Hungary; ^9^ Fejér County Szent György, University Teaching Hospital, Székesfehérvár, Hungary; ^10^ Department of Pulmonology, Petz Aladár University Teaching Hospital, Győr, Hungary; ^11^ Veszprém County Pulmonary Hospital Farkasgyepű, Farkasgyepű, Hungary; ^12^ RxTarget Ltd Szolnok, Szolnok, Hungary; ^13^ Department of Biostatistics, University of Veterinary Medicine, Budapest, Hungary; ^14^ 2nd Department of Medicine and Nephrology-Diabetes Center, University of Pécs Medical School, Pécs, Hungary; ^15^ Center for Health Technology Assessment, Semmelweis University, Budapest, Hungary; ^16^ Syreon Research Institute, Budapest, Hungary; ^17^ National Korányi Institute of Pulmonology, Budapest, Hungary

**Keywords:** lung cancer, incidence, mortality, COVID-19, Hungary

## Abstract

**Objective:**

Hungary has repeatedly been shown to have the highest cancer-related mortality and incidence in Europe. Despite lung cancer being the most abundant malignant diagnosis in Hungary, numerous concerns have been raised recently regarding the bias inherent to reported incidence estimates. Re-analysis of reimbursement claims has been suggested previously by our group as an alternative approach, offering revised figures of lung cancer incidence between 2011 and 2016. Leveraging on this methodology, we aimed at updating Hungarian lung cancer incidence estimates with an additional 5 years (2017–2021), including years affected by the COVID-19 pandemic. Additionally, we also attempted to improve the robustness of estimates by taking additional characteristics of the patient pathway into account.

**Methods:**

Lung cancer patients between 2011 and 2021 were identified based on reimbursement-associated ICD-10 codes, histology codes and time patterns. Multiple query architectures were tested for sensitivity and compared to official estimates of the Hungarian National Cancer Registry (HNCR). Epidemiological trends were estimated by Poisson-regression, corrected for age and sex.

**Results:**

A total of 89,948 lung cancer patients diagnosed in Hungary between 2011 and 2021 have been identified by our study. In 2019 alone, 7,887 patients were diagnosed according to our optimized query. ESP2013 standardized rate was estimated between 92.5/100,000 (2011) and 78.4/100,000 (2019). In 2019, standardized incidence was 106.8/100,000 for men and 59.7/100,000 for women. Up until the COVID-19 pandemic, lung cancer incidence was decreasing by 3.18% (2.1%–4.3%) yearly in men, while there was no significant decrease in women. Young age groups (40–49 and 50–59) featured the largest improvement, but women aged 60–79 are at an increasing risk for developing lung cancer. The COVID-19 pandemic resulted in a statistically significant decrease in lung cancer incidence, especially in the 50–59 age group (both sexes).

**Conclusion:**

Our results show that using an optimized approach, re-analysis of reimbursement claims yields robust estimates of lung cancer incidence. According to this approach, the incidence rate of male lung cancer is declining in Hungary, in concordance with the trend observed for lung cancer mortality. Among women aged 60–79, the incidence of lung cancer has risen, requiring more attention in the near future.

## Introduction

### Current understanding of lung cancer epidemiology in Hungary

It is widely accepted that compared to most European countries, Hungary suffers from a relatively high malignant disease incidence and mortality. This has been shown for example by a series of papers comparing 40 European countries [1–3], for both overall cancer epidemiology and individual tumor types. It is important to stress that for Hungary, these epidemiological studies estimate cancer incidence indirectly, based on statistical models relying on reported deaths and incidence in neighboring countries [4]. This indirect approach might already add some uncertainty to the estimates, while other reports have suggested mortality reported for Hungary to be exaggerated recently [5].

Furthermore, the reliability of tumor classification in reports has also been questioned by a recent amendment [6, 7] of the Hungarian National Cancer Registry (HNCR). One of the tumor types most frequently misclassified was lung cancer, possibly since lung is also a common site of metastases. Lung cancer on the other hand, is the most abundant cancer type in men, accounting for more than 20% of all cases and the top cause of cancer mortality in both sexes. Given its share, an inflated number of reported lung cancer cases can already impact the assessment of the total number of cancer patients, while the described bias might also affect other cancer types (common metastatic sites, for example), adding further uncertainty to epidemiological observations. A potential solution would be manual curation of individual records before reporting case numbers, as it was done by the HNCR with the amendment of lung cancer cases for 2018. This was found to be extremely demanding and not routinely feasible for each tumor type and year given the current resources.

### Approaches to refine incidence estimates

An alternative approach to manual curation of hospital records for the estimation of cancer incidence could be the utilization of alternative sources of information. The National Health Insurance Fund of Hungary (NHIF) is a valuable resource capturing detailed information on patient pathways via reimbursement claims. Previous publications from our research group [4, 8] have already demonstrated that epidemiological indicators can be estimated efficiently using this data source. Additionally, patient-level details captured in reimbursement claims have also provided valuable insights on the high number of *post mortem* diagnosed lung cancer cases as a potential confounder of reported case numbers [7].

Even if incidence estimated by any of the above described methods cannot be 100% accurate, reproducibility of querying the NHIF database and the possibility to finetune stringency of the query provides information on the range where the actual value lies and what biases one should look out for. This deeper understanding of epidemiological indicators is essential in the timely allocation of resources to meet emerging challenges as well as in the assessment of updates to healthcare policies. Lung cancer being the most frequent malignant disease in Hungary, it is the first choice of cancer type for modelling the robustness of epidemiological indicators and trends.

### Aims

The present research was carried out as part of the Hungarian Evaluation of Lung cancer Patient Pathway Project (HELP3) research project describing lung cancer epidemiology and patient pathways in Hungary. The primary aim of the current study is to update lung cancer incidence and mortality figures in Hungary. Recent advances uncovering potential confounders offer an excellent opportunity to refine epidemiological trends incorporating data based on the NHIF, using an improved query structure. The impact of query parameter optimization is assessed based on the robustness of trends calculated from results yielded by these queries. The impact of the COVID-19 pandemic, caused by the SARS-CoV-2 virus, on lung cancer epidemiology in Hungary can be traced in the risk ratio of pre-COVID and COVID years 2019 and 2020.

## Materials and methods

### Data sources

Yearly incidence of lung cancer was estimated based on healthcare reimbursement claims in the NHIF database, as described by our group previously [4, 8, 9]. Briefly, patients named in claims with the ICD-10 code of C34 were regarded as lung cancer patients. The year of the first occurrence of C34 was accepted as the year of diagnosis. Patients newly diagnosed between the 1st of January 2011 and the 31st of December 2021 were studied, with an additional screening period of 2009-2010 to account for patients already diagnosed outside the study period.

To further improve the specificity of our definitions, additional constraints were added to the query, specifying the number of C34 codes in an individual’s history and the minimum (30 days) as well as the maximum (180 or 365 days) amount of time accepted between two C34 codes. Patients who have died within 60 days of the first diagnosis were not required to feature multiple C34 codes in their history. This multi-tier, optimized query is referred to as “Optimized,” in sensitivity analyses (Supplementary Figure S1). When comparing the optimized query, the alternative requiring only one single mention of C33 or C34 will be referred to as *1.1*, while the query requiring 2 mentions is *1.2A*. A minimum of two mentions within a year is referred to as *1.2B*, while 2 mentions within 180 days is *1.2C*. Three mentions within 180 days is *1.3*. Estimates provided by the HNCR (stat.nrr.hu, accessed 20/10/23) were used as a reference. Please note that the optimized query is very similar to the 1.2B alternative, with the additional constraint of at least 30 days between the two claims and a small relaxation regarding patients deceased within 60 days.

Number of deaths associated with lung cancer are based on reports of the Hungarian Central Statistical Office (HCSO). Both incidence and mortality were standardized using the European Standard Population (ESP) of 2013 and standardized rates are given using that reference, unless otherwise indicated. For the sake of comparison with estimates of Ferlay’s workgroup, rates standardized to the ESP 1976 population are also shown in this context. To facilitate reproducibility of our results, patient numbers yielded by NHIF queries, retrieved from the HNCR or the HCSO are provided in a tidy long format as Supplementary Table S1, as well as mid-year population retrieved from the HCSO (Supplementary Table S2)

### Statistical analysis

All calculations were carried out in the open source software environment for statistical computing R (v4.2.1). Average Yearly Change was estimated by Poisson-regression, correcting the model for population size, age, and sex. The logarithmic value of the number of individuals in a given age group was used as offset, while age, sex and year were explanatory variables. Statistical significance was inferred from the *p*-values of Wald-tests implemented by the *Testing Linear Regression Models* (lmtest v0.9) package and considered significant in case it was *p* ≤ 0.05. Confidence intervals (95% CI) were estimated using the packages lmtest and *Robust Covariance Matrix Estimators* (sandwich v3.1) variance estimation [10] to correct for non-independent nature of the data. A normal distribution was assumed when assigning (95% CI) of direct standardized rates. Risk ratio was calculated using the *Epidemiology Tools* (epitools v0.5) package.

## Results

### Annual number of patients

The number of yearly diagnosed lung cancer patients in Hungary ranged between 8,752 and 7,003. For 2019, the last year before the COVID-19 pandemic, our query after optimizations has identified 7,887 new lung cancer patients (Supplementary Figure S1). As a comparison, the least stringent case definition 1.1 returned 9,600 hits, while changing the stringency by requiring multiple visits in the patient history (filters 1.2A, 1.2B, 1.2C) increased the number of candidates to 8,498, 8,144 and 8,240, respectively. Requiring 3 visits, without further restrictions (filter 1.3C) picked up only 7,121 potential lung cancer cases.

The highest number following each approach was observed for 2011 and a steady decrease in patient numbers could be observed until 2020, featuring the lowest number of new patients during the studied period. For each year, a male predominance in the number of patients could be observed. For the initial year 2011, 5,517 men and 3,235 women were found to be diagnosed with lung cancer. By the end of the study period, this difference was smaller (4,424 and 3,436, respectively, in 2019), but still visible.

### Age standardized rates

**FIGURE 1:**
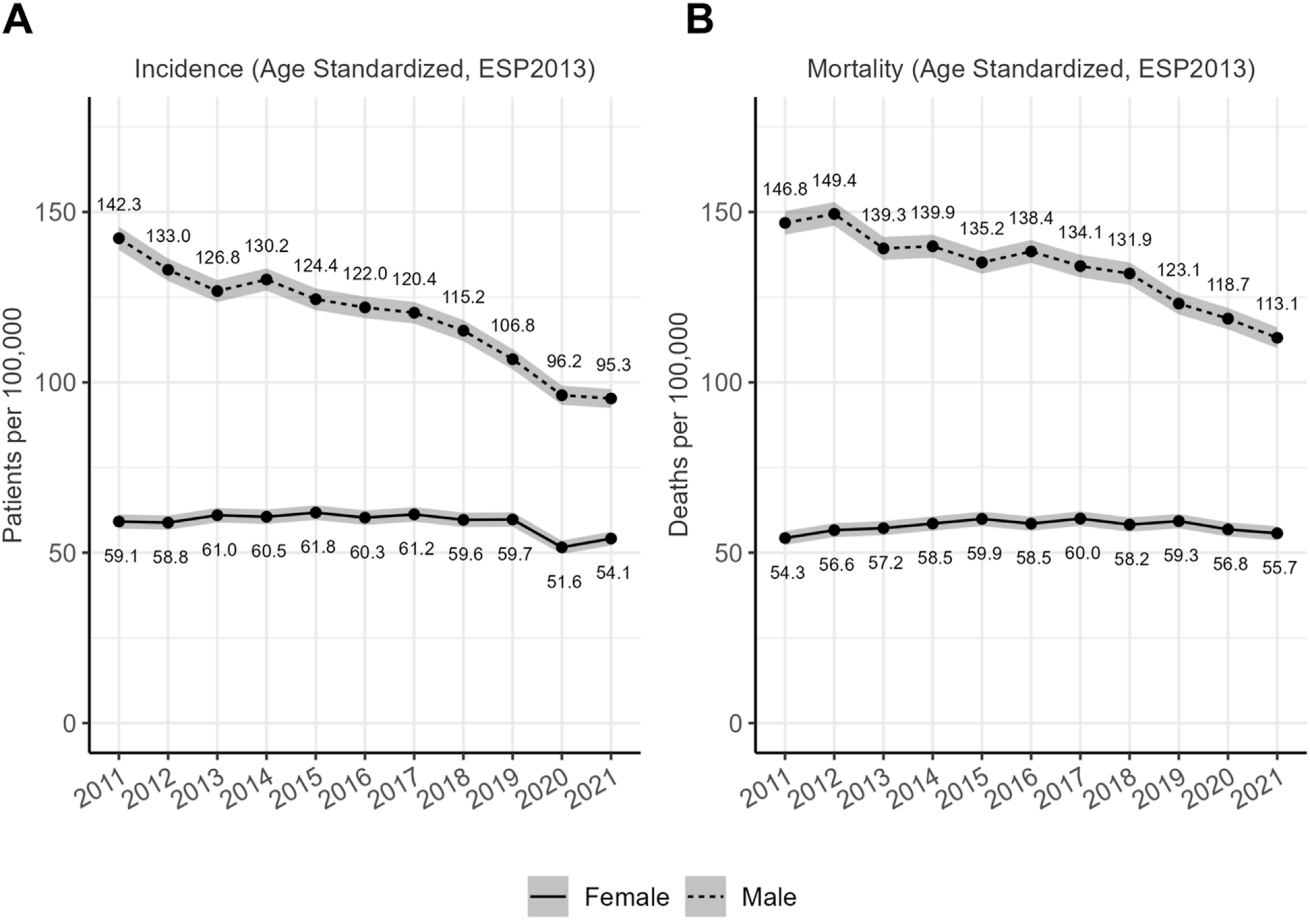
**(A)** Standardized incidence rates of lung cancer in Hungary between 2011 and 2021. Direct standardized rates using the ESP2013 population weights. Grey ribbon around the lines represents the 95% confidence interval. **(B)** Standardized mortality rates of lung cancer in Hungary between 2011 and 2021 (ESP2013 standardized rates). Direct standardized rates using the ESP2013 population weights. Grey ribbon around the lines represents the 95% confidence interval.

In 2011, according to the results of the optimized, consensus query, there were 142.3/100,000 (ESP2013) new male lung cancer patients identified, while only 59.1/100,000 (ESP2013) female patients (Figure 1A). Standardized incidence has decreased to 106.8/100,000 in men by 2019, while it remained at the same level with 59.7/100,000 for women. As a comparison, standardized mortality due to lung cancer was reported to be 146.8/100,000 in men and 54.3/100,000 for women (Figure 1B) in 2011, which has changed to 123.1 in men and 59.3 in women. A detailed report of standardized incidence (Supplementary Table S3) and mortality (Supplementary Table S4) rates for all studied years and both sexes can be found in the Supplementary Material.

**FIGURE 2:**
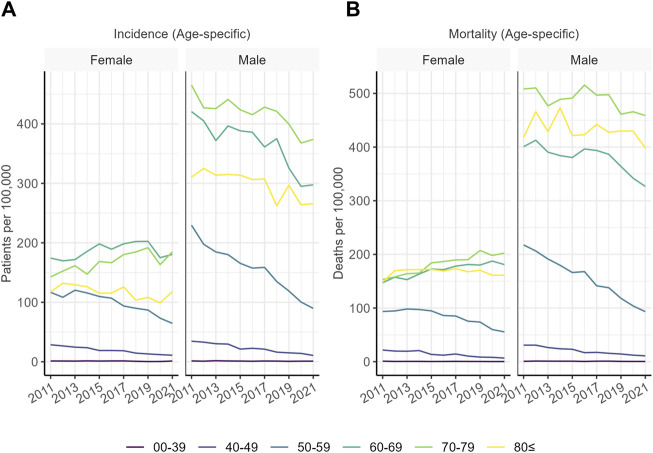
**(A)** Age-specific incidence of lung cancer in Hungary in the studied age groups. Values indicate the number of patients diagnosed in a given year out of 100,000 individuals of the age group. **(B)** Age-specific mortality of lung cancer in Hungary in the studied age groups. Values indicate the number of patients deceased in a given year out of 100,000 individuals of the age group.

Lung cancer had the highest incidence in the 60–69 (women) and 70–79 (men) age groups (Figure 2). Age distribution of lung cancer patients has been shifting towards stronger representation of older age groups, especially in women. Age distribution of patients died of lung cancer shows a similar pattern, with an even more pronounced decrease in the share of the 40–49 age group in both sexes.

### Observed trends

**FIGURE 3:**
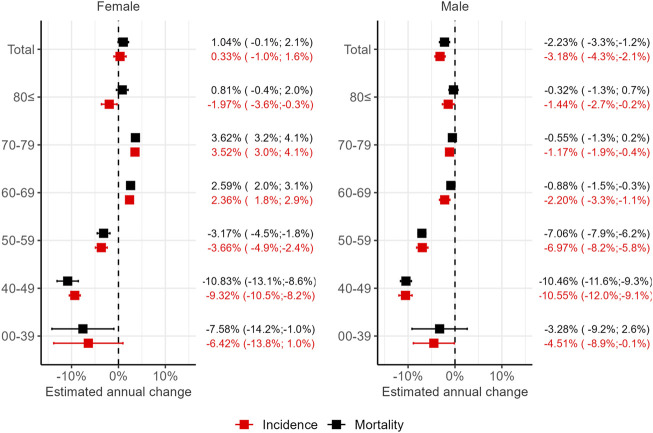
The average annual change in lung cancer incidence and mortality in Hungary between 2011 and 2019 for the total population, as well as by age groups. Estimated using Poisson regression and robust confidence intervals (95% CI) with the sandwich method.

Lung cancer incidence in the general population was found to decrease between 2011 and 2019 by 1.76% on an annual basis (95% CI: 0.5%–3%). For men only, the decrease was 3.18% (95% CI: 2.1%–4.3%), while for women, the estimated 0.33% increase (95% CI: −0.1%–1.6%) did not indicate a significant change (Figure 3; Supplementary Table S7). Young age groups (40–49 ad 50–59) featured the largest decrease in lung cancer incidence for both women and men. Conversely, the incidence was less likely to decrease in the 60–69 and the 70–79 age groups. Lung cancer incidence in elderly women has even increased slightly during the study period.

A similar pattern of lung cancer mortality (Figure 3; Supplementary Table S8) could be observed between 2011 and 2019, with an overall decrease of 0.99% (95% CI: −2.0%–0.1% change). While the overall trend was not found to be statistically significant, there was a significant, 2.23% decrease (95% CI: −1.2%–3.3%) change when taking only men into account. The 1.04% yearly increase (95% CI: −0.1%–2.1%) estimated for women was not statistically significant either. An improvement in mortality could be confirmed in the 40–49 and 50–59 age groups of both sexes, while mortality has increased among women aged 60–69 and 70–79.

### COVID-19 impact

**FIGURE 4:**
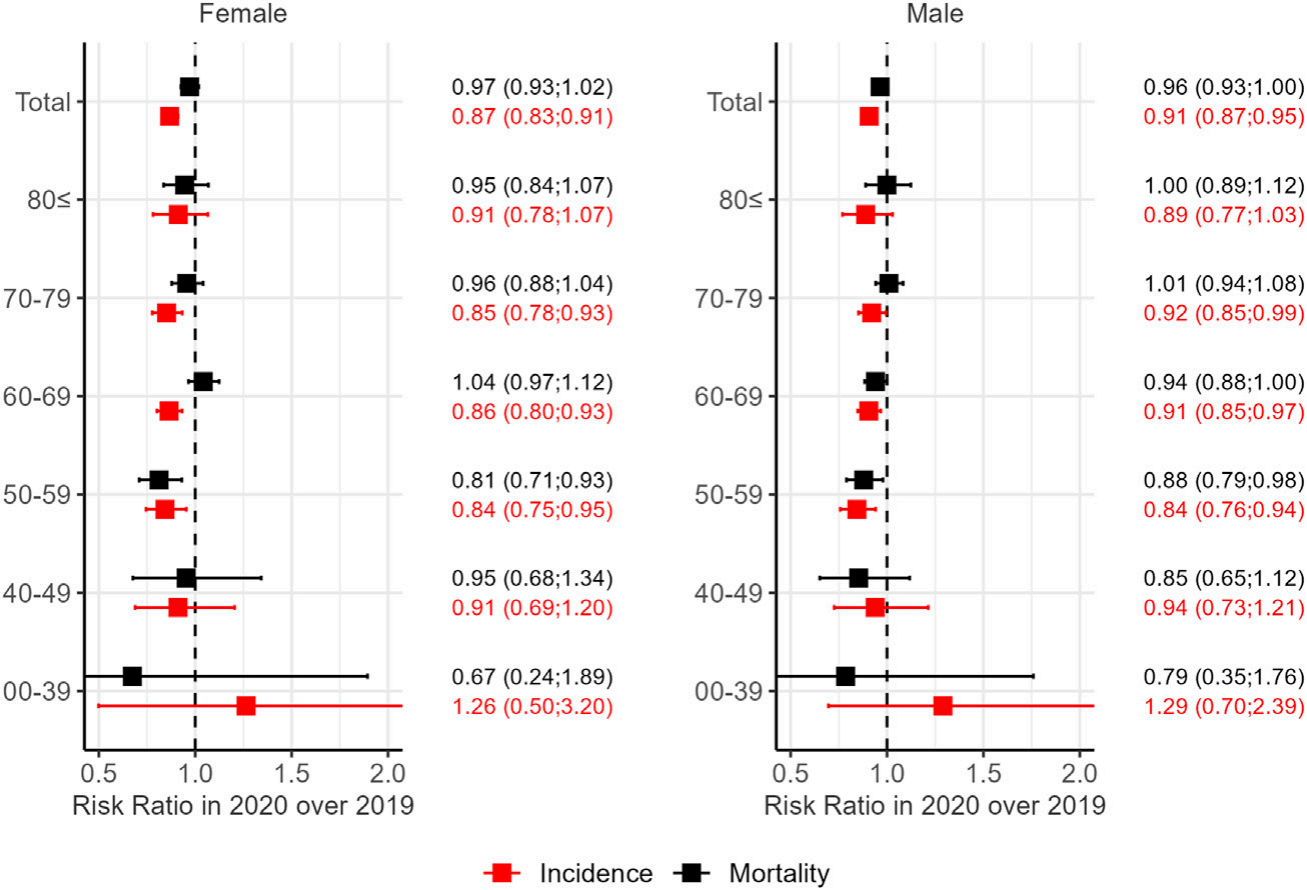
Change in lung cancer incidence and mortality in Hungary during the COVID year 2020 expressed as the risk ratio (RR) of 2020 vs. 2019. Confidence intervals (95% CI) of RR are given in brackets.

To assess the impact of the COVID-19 pandemic on lung cancer incidence and mortality in Hungary, the pandemic year 2020 was compared to the previous year, 2019 (Figure 4). The risk of lung cancer diagnosis was significantly lower for both women and men in 2020 than in 2019 (Risk Ratio (RR) = 0.87 and 0.91, respectively), while the risk of lung cancer specific death did not decrease significantly (RR = 0.97 and RR = 0.96, respectively). The difference in terms of patient numbers means that 891 less patients were diagnosed with lung cancer in 2020 than in 2019 (6,970 and 7,861, respectively). The decrease in incidence compared to 2019 was driven mainly by the 50–59, 60–69, and 70–79 age groups in both sexes. Lung cancer mortality, however, showed a similar decrease only in the 50–59 age group (RR = 0.81, women; RR = 0.88, men). The calculated RR values for incidence (Supplementary Table S9) and mortality (Supplementary Table S10) are also available in the Supplementary Material.

### Sensitivity analysis

Standardized lung cancer incidence estimated using different case definitions resulted in a range around 133–179/100,000 for men and a narrower range of 55–79/100,000 for women in 2011 (Supplementary Figure S2). Estimate using the less stringent query (1.1) was close to the number reported by the HNCR and restricting the definition by requiring more than one record featuring the ICD-10 code of lung cancer (1.2A) already yielded results similar to the final estimate. Restrictions regarding the minimum or maximum amount of time between visits (1.2B or 1.2C) had a much smaller impact on the results. Capturing the raw numbers behind these rates, a total of 8,191 to 11,261 new lung cancer patients were found for the reference year 2012 (Supplementary Figure S1). The least stringent case definition aligns well with the 11,000 patients reported by the HNCR. Using different case definitions had hardly any effect on the time trend analysis (Supplementary Figure S3).

### European context

Comparison of previously reported standardized incidence rates of Hungary to neighboring countries also suggests a possible bias, resulting in the overestimation of Hungarian lung cancer incidence. In a comprehensive study including 40 European countries, Ferlay’s workgroup has reported a 109.3/100,000 male lung cancer incidence rate (ESP1976) for Hungary in 2012 [3] and 111.6/100,000 in 2018 [2]. These rates would be extremely high in Europe and this extent of outlying alone would be alarming. On the other hand, the 133 per 100,000 Person Years estimated based on the ESP2013 standards for 2012 and reported above (Figure 1.), is equivalent to 95.2/100,000 calculated using the ESP1976 weights. Similarly, the ESP1976-eqivalent incidence rate for 2018 was 80.5/100, 000 based on our data. These estimates are much closer to the (Central) European average (Supplementary Figures S4, S5) than the ones reported previously for Hungary. The ESP1976 standardized rates for all studied years are provided for multiple incidence estimates (Supplementary Table S5) as well as for mortality reported by the HCSO (Supplementary Table S6) in the Supplementary Material.

## Discussion

### Updated lung cancer incidence in Hungary

By extending the studied period to more than 10 years, the current study provides updates to epidemiological indicators of lung cancer in Hungary with an even more refined approach, but also largely building on our previous reports [4, 7, 9]. This update confirms the inconsistency between lung cancer incidence rates reported previously for Hungary and the rates estimated by our approach. Differences in the numerical values of lung cancer estimates reported for Hungary are partially due to differences in standardization methods as they have been evolving through time. Values given relative to the ESP2013 population can be converted to reflect the rate in the ESP1976 population, as also done in this study to compare with historical data. Even after conversion, however, conflicting rates have been for Hungary [2, 3]. We claim that this remaining difference originates at least partially from differences between statistical models used to estimate incidence. One can even trace the changes that occur to the model used by one single research group over time, changing the input slightly to rely on different countries [2, 3], to improve confidence of the estimate.

Appreciating that the increasing weight of input from the national cancer registry of the Czech Republic in the reported Hungarian estimate is in agreement with our observations regarding the similarity of healthcare indicators in Hungary to other Eastern European countries [7], we would like to advocate for granting greater importance to local datasets, when developing these models. Since the availability of observational data for Hungary is limited currently, we would like to provide such empirical data, hoping to contribute to closing the gap between lung cancer incidence estimates in the literature.

While a critical revision of standard incidence contributes to a realistic assessment of current disease burden, the trends described by these figures are even more crucial when planning future preventive actions. Acknowledging that limitations to our approach prevent us from identifying some patients, thus our data can be regarded as close estimates only, we argue that the trends identified in these figures are robust enough to identify emerging needs or give positive feedback on improvement. Differences in the dynamics of the epidemiology of lung cancer between women and men has been shown multiple times, including our publications [4], and our current results recapitulate this pattern nicely. Furthermore, an increasing lung cancer incidence among women between 60 and 79 already points out a population that needs more attention regarding prevention, early detection, or care.

### Improving trend in 2011–2019

Our data suggests that lung cancer incidence has started to decline during the last decade (2011–2019) in men, similar to observations for Germany [11]. This observation is in line with our previous reports on lung cancer, describing improvements over previous periods [4, 8], in contrast to interpretations forecasting an alarming number of new lung cancer cases [12]. As smoking contributes to about 85%–90% of lung cancers [13], based on the slowly decreasing smoking prevalence in almost every European country [14], a consequent decrease in lung cancer suggested by our results seems logical.

An increase in lung cancer incidence within the population would only be expected if other risk factors were emerging, or in certain subpopulations. While air pollution could be such an emerging risk factor, showing a well-established association with lung cancer etiology [15], no major rise in Particulate Matter <2.5 µm (PM2.5) or PM10 levels has been reported for Hungary during the study period. Nevertheless, PM2.5 levels, a recently re-confirmed etiological factor in the pathogenesis of adenocarcinoma [16], have been constantly high in Hungary for decades [17]. This prolonged exposition might contribute to the increased risk among women. Occupational risk factors, such as asbestos have been shown to contribute little to population-level lung cancer risk in Central Eastern Europe [18]. Inherited genetic variations generally contribute to lung cancer etiology indirectly via susceptibility to environmental exposure [13]. Even though positive family history of lung cancer has been shown to increase lung cancer risk in the Eastern European population [19], contribution of genetic causes can still be considered minor compared to the risk of smoking in patients above 50.

While the observed improvement at the population level is logical considering the decrease in smoking prevalence, the lack of improvement among women is in certain age groups is somewhat surprising. As described in our previous revision of lung cancer epidemiology in Hungary [4], however, smoking prevalence is not homogenous in the population and even gender imbalance has dynamically changed over time. In contrast to men, where the number of ever-smokers has decreased steadily in every birth cohort, an increase in smoking prevalence has preceded the recent decrease in women. This difference in smoking patterns has already been shown to affect lung cancer incidence in European populations [20] and explains the heterogenous trends observed in our data stratified by age groups.

It is also important to note that our study focuses on changes regarding the risk of an individual, captured in standardized incidence and mortality rates. Standardization is carried out precisely to compensate for the confounding effect of the population’s aging. The increasing number of elderly people in a population inevitably leads to an increase in age-related diseases, like lung cancer. This might lead to a paradoxical observation of increasing patient numbers (and burden on the healthcare system) even when the individual’s risk is decreasing. Although both indicators (patient numbers and risk) carry important information, we decided to focus on risk as this better describes the effect of preventive measures or the improvement in quality of care. Nonetheless, this is the measure that can be compared among countries or regions.

### The impact of COVID-19

The COVID-19 pandemic declared by the World Health Organization (WHO) in 2020 has resulted in a sudden, dramatic change in accessibility. The impact of these restrictions on lung cancer detection has been shown for example by a direct comparison of the number of lung cancer cases in the UK during lockdowns in 2020 and the same period of 2019 [21], reporting a 26% decrease during the pandemic. A 14.4% decrease in lung cancer incidence has also been observed for Hungary, together with decrease in breast and colorectal cancer patient numbers [22]. Thus, the incidence during the COVID-19 pandemic was not specific to lung cancer, only the extent of the decrease varied between tumor types. In addition to confirming the decreased breast cancer incidence [23], we have also described that breast cancer mortality did not increase significantly. This suggests that care of patients already diagnosed was not affected by restrictions or bottlenecks in resources, but rather the detection of cancer cases was delayed. Screening program participation, for example, has also reduced dramatically due to the pandemic: 25.8% less centrally organized mammography examinations were conducted in 2020 than in the previous year [24]. The reduction in diagnostic capacities was probably even greater in the case of cancer types where an organized screening program is not available, like lung cancer.

Reduced incidence was not associated with a change in mortality in our study population of Hungarian lung cancer patients, similarly to what has been described for breast cancer earlier [23]. Delayed diagnosis is, however, expected to cause an increase in lung cancer mortality of around 4.8%–5.3% during the next years, as suggested by a modeling study based on the UK population [25]. Another model based on the Australian population warns about a potential mid-term reversal of positive epidemiological trends (decreasing cancer incidence and mortality) caused by the aftermath of the restrictions in 2020 [26].

Patients not identified in 2020 are also likely to increase lung cancer incidence or at least mortality in the post-pandemic era. According to our data, as many as 891 lung cancer patients might have been missed in 2020. Even if the decreasing trend is considered, ∼800 lung cancer patients will receive a delayed diagnosis due to the COVID-19 pandemic. These patients are expected to either show up in the healthcare system as an excess number of new patients in 2021, 2022 or might never actually be identified if they also suffered from COVID and the outcome was fatal. It is not yet clear from currently available data, which scenario is true, but these considerations should be taken into account, when assessing lung cancer epidemiology of post-pandemic years.

Lung cancer incidence has decreased during the COVID-19 pandemic primarily in the 50–59, 60–69, and 70–79 age groups, both sexes. The sex-related pattern of lung cancer epidemiology is not reflected in the changes attributable to the COVID-19 pandemic. Interestingly, no decrease was seen among elderly patients, above 80. This might be due to the simultaneous prevalence of multiple diseases in this age group and consequently, frequent hospitalizations. There are sporadic reports on incidental findings on chest CTs requested to confirm COVID turned out to be early lung cancer [27] and these non-targeted diagnostic procedures might have been carried out at a higher rate in elderly, multimorbid patients presenting with symptoms suggestive of COVID. Even if part of the population at risk for lung cancer might have profited from the preventive measures during the pandemic, the chance of recognizing lung cancer was hindered in the general population.

The only age group featuring a significant change in mortality is patients between 50 and 59. Our data is not sufficient yet to decide if this age group profited from the alertness of the healthcare system during the pandemic or this decrease is independent of the COVID-19 pandemic. The strongly decreasing trend in both incidence and mortality of lung cancer in this age group already before the COVID-19 pandemic suggests an idiosyncratic effect.

### Robustness of data and limitations

A major limitation to our claims-data-based approach is that detailed clinical history is not available in this database, thus it is not possible to medically validate the records. In fact, this confirmation would also not be feasible at this scale even if all health records were available and accessible. In a similar attempt, the HNCR carried out manual validation of the diagnoses associated with every individual reported as lung cancer patients in 2018 [28]. Reviewing the patients from one single year has already proven to be a tremendous effort, far beyond resources available for epidemiological studies. The amended patient number (9,541 patients), however, is an important reference to benchmark any alternative approach, such as the one followed in this study.

The present approach has identified 8,252 patients in 2018 that is lower than the reference reported by the HNCR. Identifying this subset of claimed patients with a high probability of a valid disease classification might offer an opportunity on the other hand to associate a confidence level to patients registered by the HNCR. Despite still not being able to identify some lung cancer patients, performance of the current approach features remarkable improvements even over the method used in a previous study based on the same source [4]. This improvement was enabled by a combinatorial optimization of the query parameters, better adapting to common patient pathway scenarios. One such scenario would be the common case, when a late-stage patient is not able to go through the diagnostic procedure to arrive at a definite diagnosis. By requiring less stringent conditions for patients deceased within 60 days of the first claim featuring a lung cancer related ICD-10 code, reduces the risk of missing these patients. Using patient pathway-related criteria to exclude patients coded as lung cancer by mistake is a reproducible, high-throughput approach that is feasible to be carried out even in case of large patient cohorts. Thereby we suggest that, while not being able to achieve data quality offered by manual curation, it is able to flag potentially miscoded patients; the error type identified most frequently during manual curation [28].

Another potential issue identified by researchers at the HNCR, could be the inconsistent reporting of lung metastases from other sites [5] and this is related to a third source of uncertainty, identified by our research group recently: the considerable number of lung cancer patients diagnosed *post mortem* only [7]. As a comparison, ∼10% of lung cancer cases in Germany are recognized only during autopsy [11]. Patients presenting at a very advanced stage might be in such a severe condition already that diagnostic procedures cannot be carried out and a definite diagnosis will never be made. Although some of these patients might appear in the claims dataset under ICD-10 codes D38 or R91H0, it is very hard to assess the number of the patients recognized at this stage and we cannot describe the change in this patient segment over the studied period using our method. A change in coding preference of the hospitals during the period might also confound our observations. Both lung metastases and *post mortem* diagnosed patients add a further level of complexity to obtaining a realistic estimate of lung cancer incidence, but a consistent, reproducible methodology described here can still provide valuable insights on epidemiological indicators.

Bias related to *post mortem* diagnosis, or even unspecified metastases could also impact on cause-specific mortality reported by the HCSO. It has already been proposed by studies conducted within the HNCR that a slight overestimation of patient numbers is inherent to many cancer registries [5] and perhaps, even mortality reports. According to our previous observations described in the HULC study [7], the number of *post mortem* diagnosed lung cancer cases is comparable to the total number of lung cancer patients. Based on the proportion of these cases, it seems logical that individuals never diagnosed with lung cancer, but reported after an autopsy to have died of the disease, contribute to an overestimation of lung cancer mortality, as suggested also by the HNCR. A further argument supporting this possibility is that the mortality-to-incidence ratio (MIR) calculated from our revised incidence rates is close to 1, while globally it has been around 0.7 [29, 30]. This problem does not affect every cancer type equally. Lung cancer is likely among the sites more severely impacted by this bias due to the high probability of metastases from other sites developing in the lung. Similarly, not every country is affected equally, possibly due to the disparate autopsy rates among countries, as discussed earlier [7]. While revising the accuracy of diagnosis for patients registered in the HNCR has been carried out recently [28], such a revision regarding cause-specific mortality reported by the HCSO was not in the scope of the current study.

Regardless of the number of lung cancer patients missed by our approach, it is important that the error rate is constant across years, rendering trend estimations based on our data robust and reproducible. As shown in Supplementary Figure S3, the stringency of our case definition did not significantly change the trend estimated by our models. As an alternative validation approach, incidence trends estimated by our approach were also compared to mortality trends. Only one age group (women above 80) featured a significant difference that confirms the expected parallel change of incidence and mortality in most age groups. Notably, trend estimated based of HNCR data is also not parallel with mortality in this single age group. These observations support the validity of epidemiological trends calculated using our approach and corroborates evidence supporting recent improvements in Hungarian lung cancer epidemiology.

## Conclusion

Our results provide further evidence challenging currently reported case numbers and offers an alternative approach to estimate lung cancer incidence, yielding more robust estimates. Better alignment of the resulting figures with numbers reported from neighboring countries, as well as with the dynamics of cause-specific mortality suggest that the more conservative estimates are closer to the actual number of new patients. Without denying the burden imposed by lung cancer on Hungary, we would like to advocate for a more realistic picture, where the dynamics of lung cancer in Hungary is very similar to other Central European countries.

A more realistic assessment of lung cancer epidemiology in Hungary offers a better opportunity to identify emerging challenges, allocate resources and confirm success. Furthermore, our data suggests that the described trends are even more robust than the individual yearly estimates. Identifying differences in the dynamics of the epidemiology between women and men offers opportunities for intervention. One such emerging challenge identified by our study is the increasing risk of lung cancer in women between 60 and 79 despite improvements in the general population. Robust estimation of lung cancer incidence also contributes to a more precise assessment of the impact of the COVID-19 pandemic on delaying lung cancer diagnosis and highlight potential consequences.

## Data Availability

The original contributions presented in the study are included in the article/Supplementary Material, further inquiries can be directed to the corresponding author.

## Supplementary material

SUPPLEMENTARY FIGURE S1 Comparison of patient numbers estimated via different approaches. The consensus estimate of the present study is compared to the raw number of incident patients reported by the National Cancer Registry (NCR) as well as to query variations (1.1–1.3C, see methods for detailed definition) featuring different levels of stringency.

SUPPLEMENTARY FIGURE S2 Sensitivity analysis of incidence estimates based on different approaches. Direct standardized rates are calculated for the ESP2013 population standard. The consensus estimate of the present study is compared to the incidence reported by the National Cancer Registry (NCR) as well as to query variations (1.1–1.3C, see methods for detailed definition) featuring different levels of stringency.

SUPPLEMENTARY FIGURE S3 Sensitivity analysis of trend estimations. Epidemiological trends estimated based on officially reported mortality, reported new patients in the National Cancer Registry (NCR) and queries of different stringency (1.1–1.3C, see methods for detailed definition) are compared to the consensus estimated by the present study. Annual change estimated using Poisson regression; 95% confidence intervals calculated via the sandwich method. Young age cohorts with small numbers of reported cases not shown for the sake of clarity.

SUPPLEMENTARY FIGURE S4 Hungarian lung cancer incidence (women) in the European context. Female Lung cancer incidence rates standardized to the ESP1976 population by our approach for 2012 and 2018 (Hungary*) are compared to numbers reported by Ferlay et al. for Hungary, as well as other European countries.

SUPPLEMENTARY FIGURE S5 Hungarian lung cancer incidence (men) in the European context. Male Lung cancer incidence rates standardized to the ESP1976 population by our approach for 2012 and 2018 (Hungary*) are compared to numbers reported by Ferlay et al. for Hungary, as well as other European countries.
