# Supplementary figures and images for: Decreasing incidence and mortality of lung cancer in Hungary between 2011 and 2021 revealed by robust estimates reconciling multiple data sources
Source: Pathol Oncol Res. 2024 Jun 3;30:1611754. doi: 10.3389/pore.2024.1611754 (PMC11181153; doi:10.3389/pore.2024.1611754)

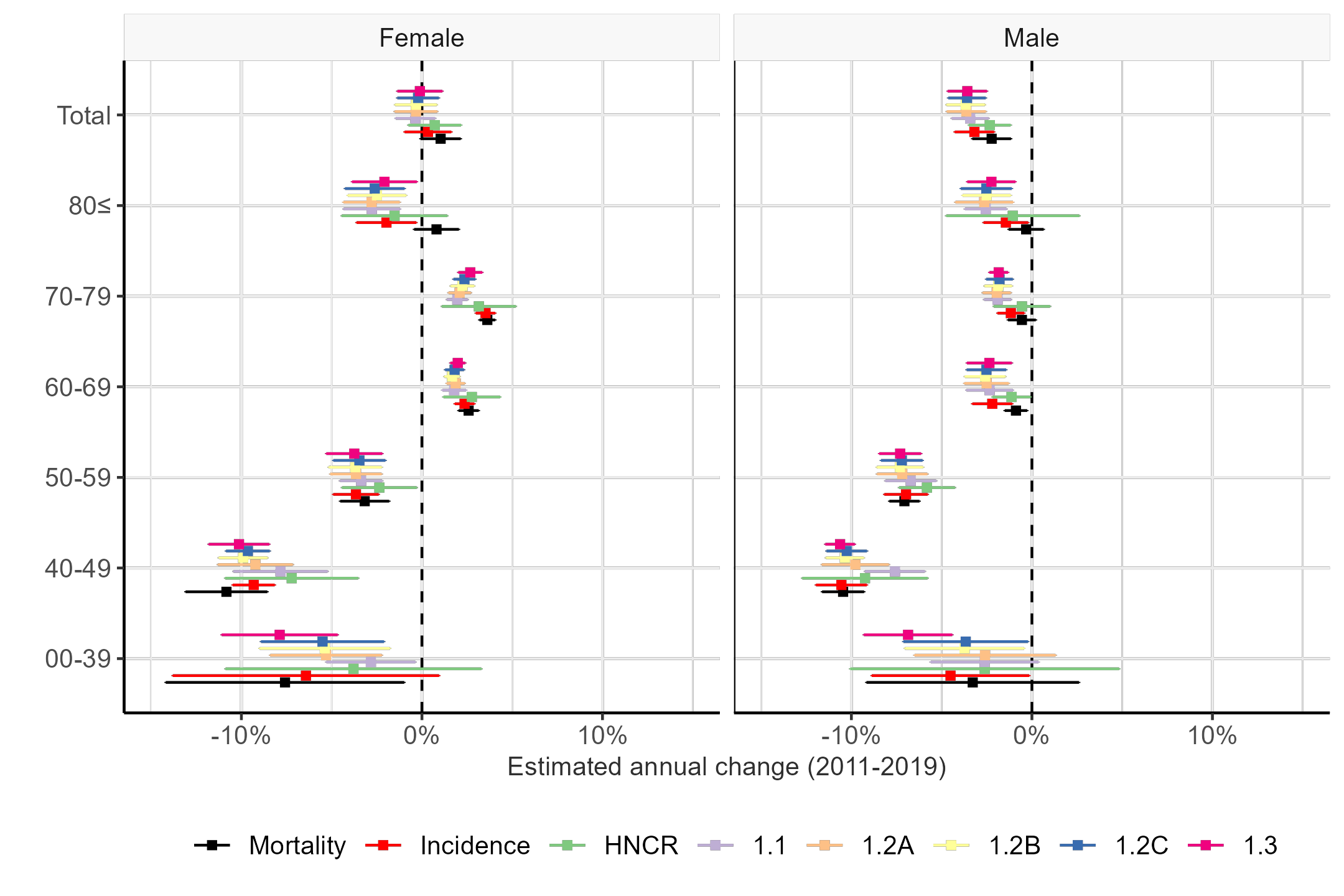

Supplement: Supplementary file 1 [file Image3.TIFF]

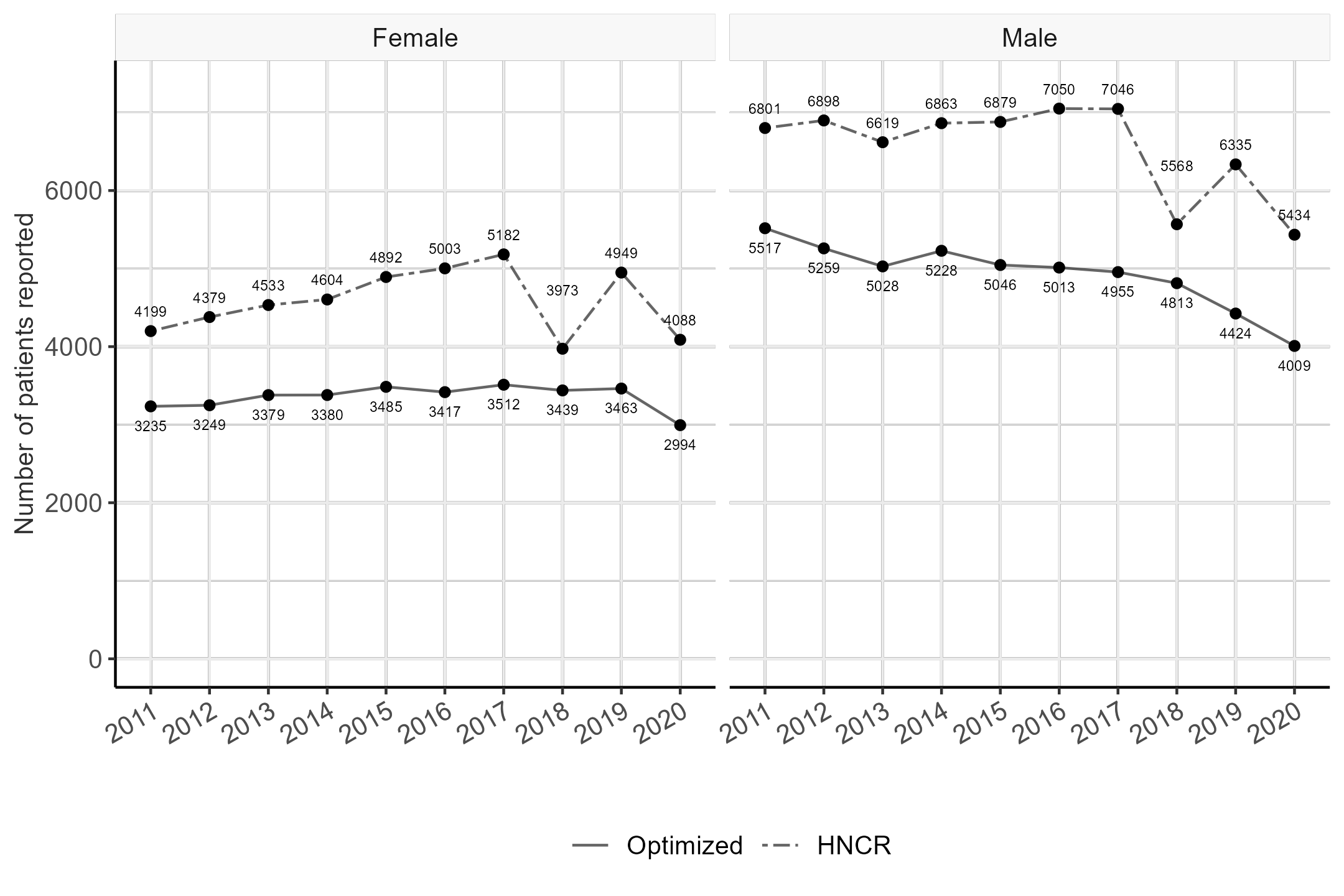

Supplement: Supplementary file 2 [file Image1.TIFF]

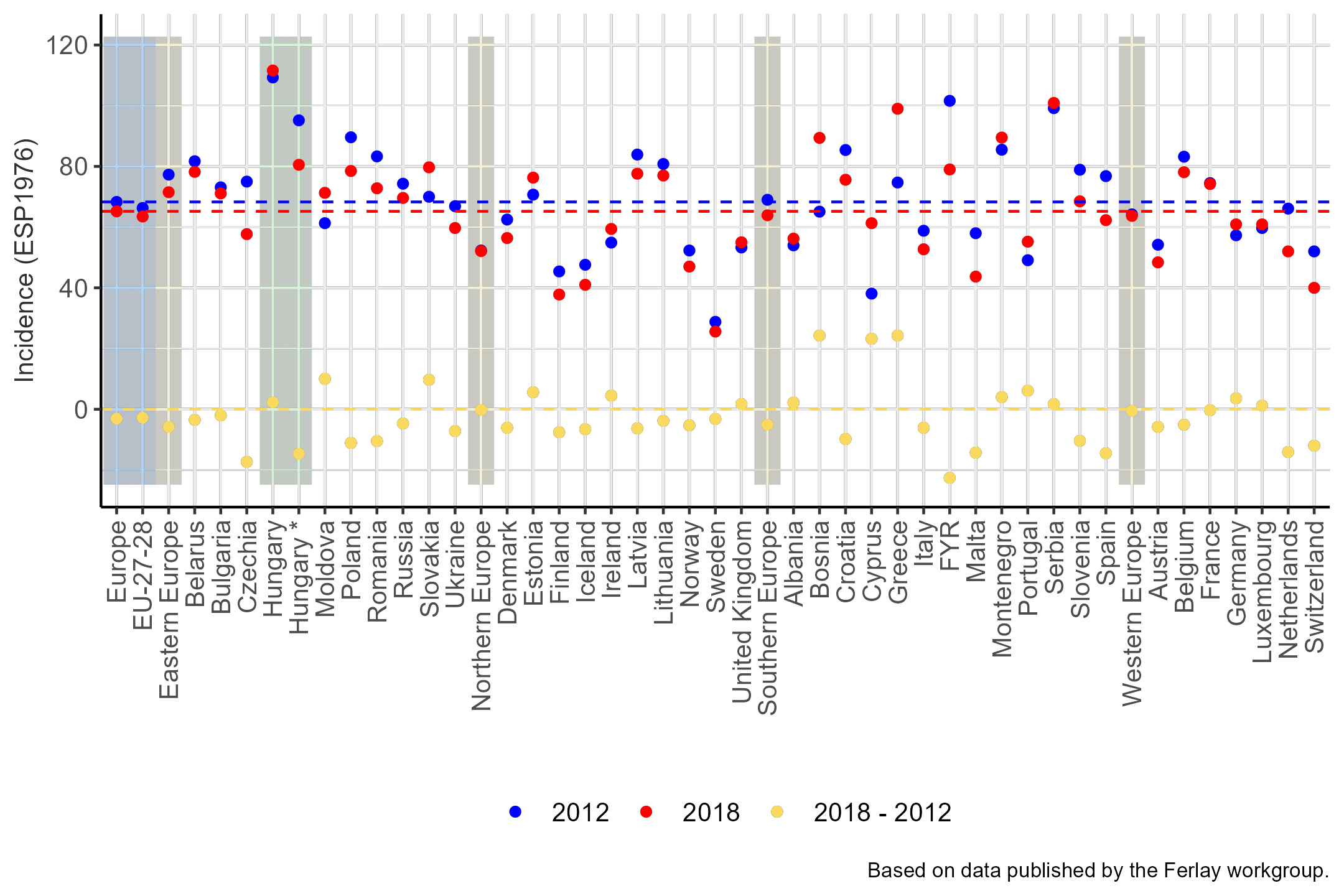

Supplement: Supplementary file 3 [file Image5.TIFF]

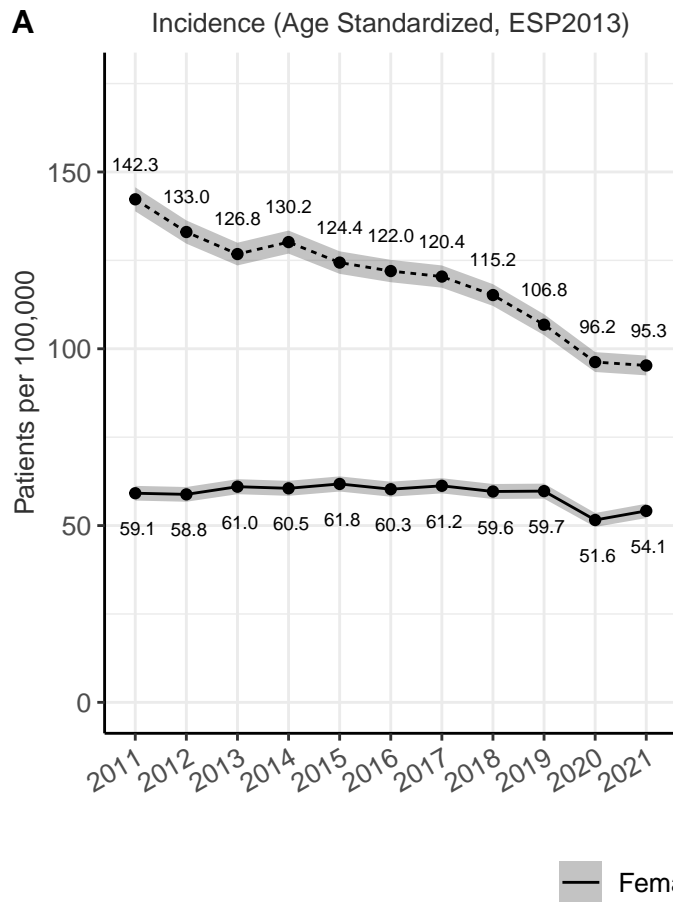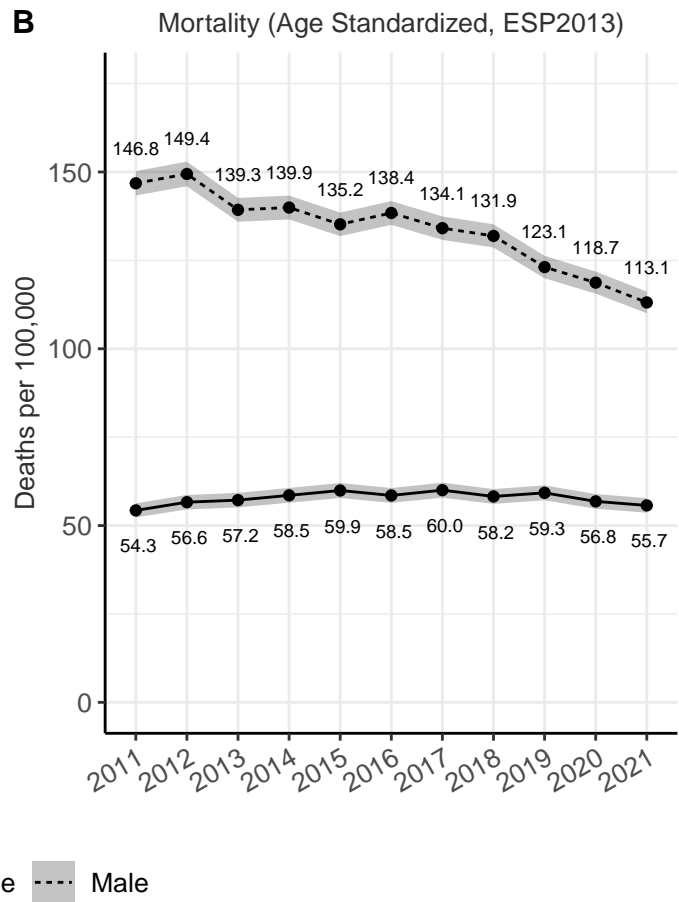

Supplement: Supplementary file 4 [file DataSheet1.ZIP › figures/fig1.pdf]

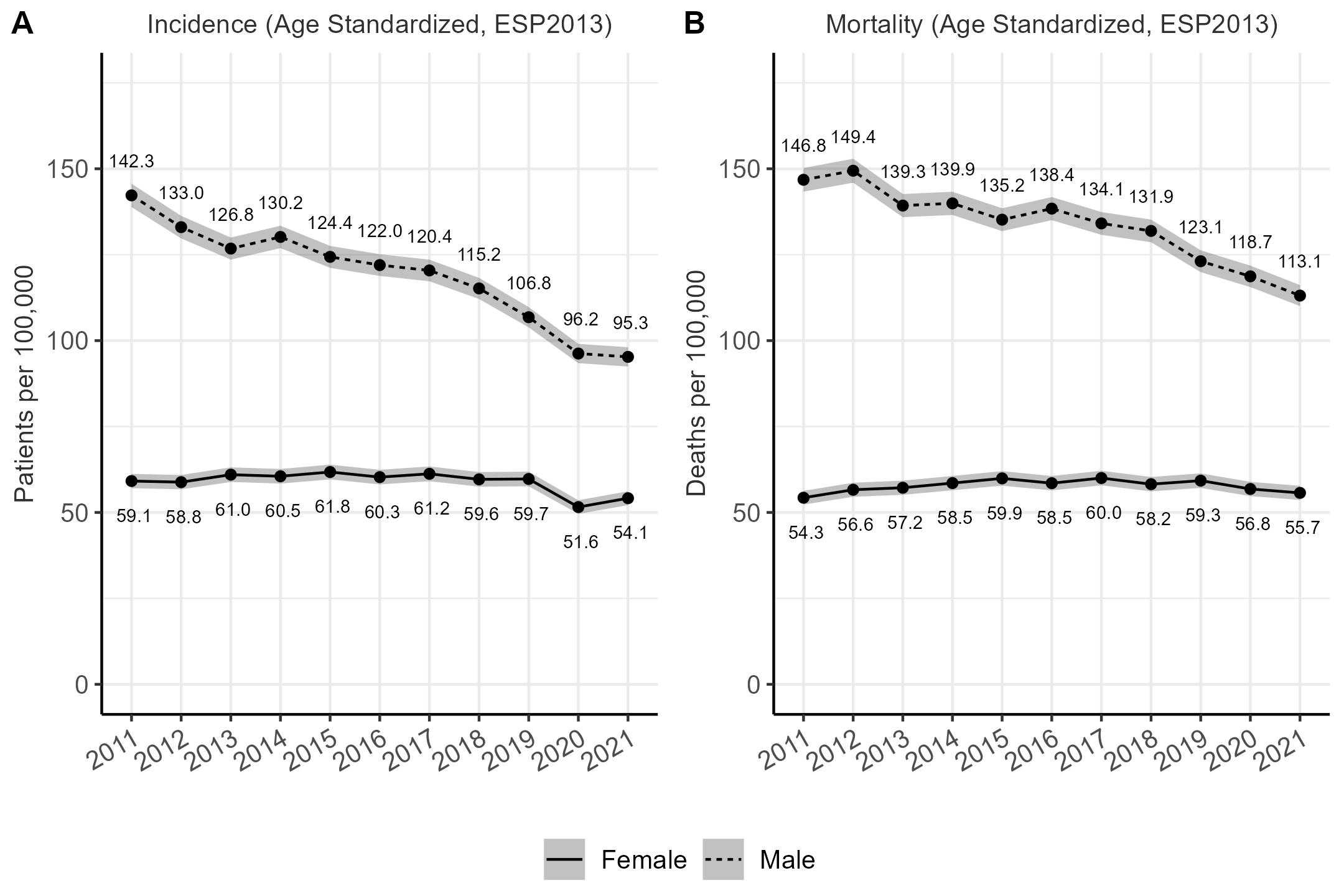

Supplement: Supplementary file 4 [file DataSheet1.ZIP › figures/fig1.png]

**A**

Incidence (Age-specific)

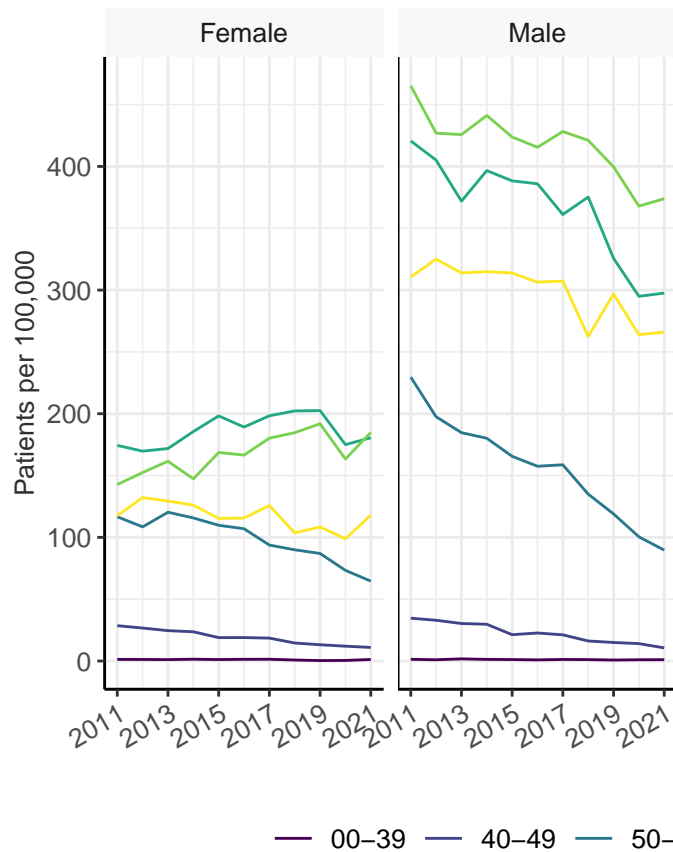**B**

Mortality (Age-specific)

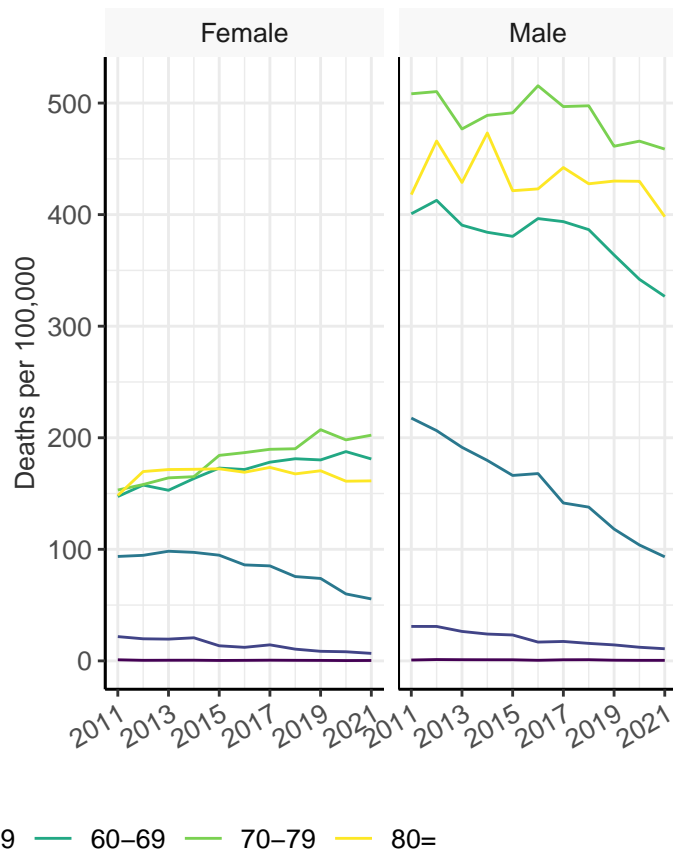

Supplement: Supplementary file 4 [file DataSheet1.ZIP › figures/fig2.pdf]

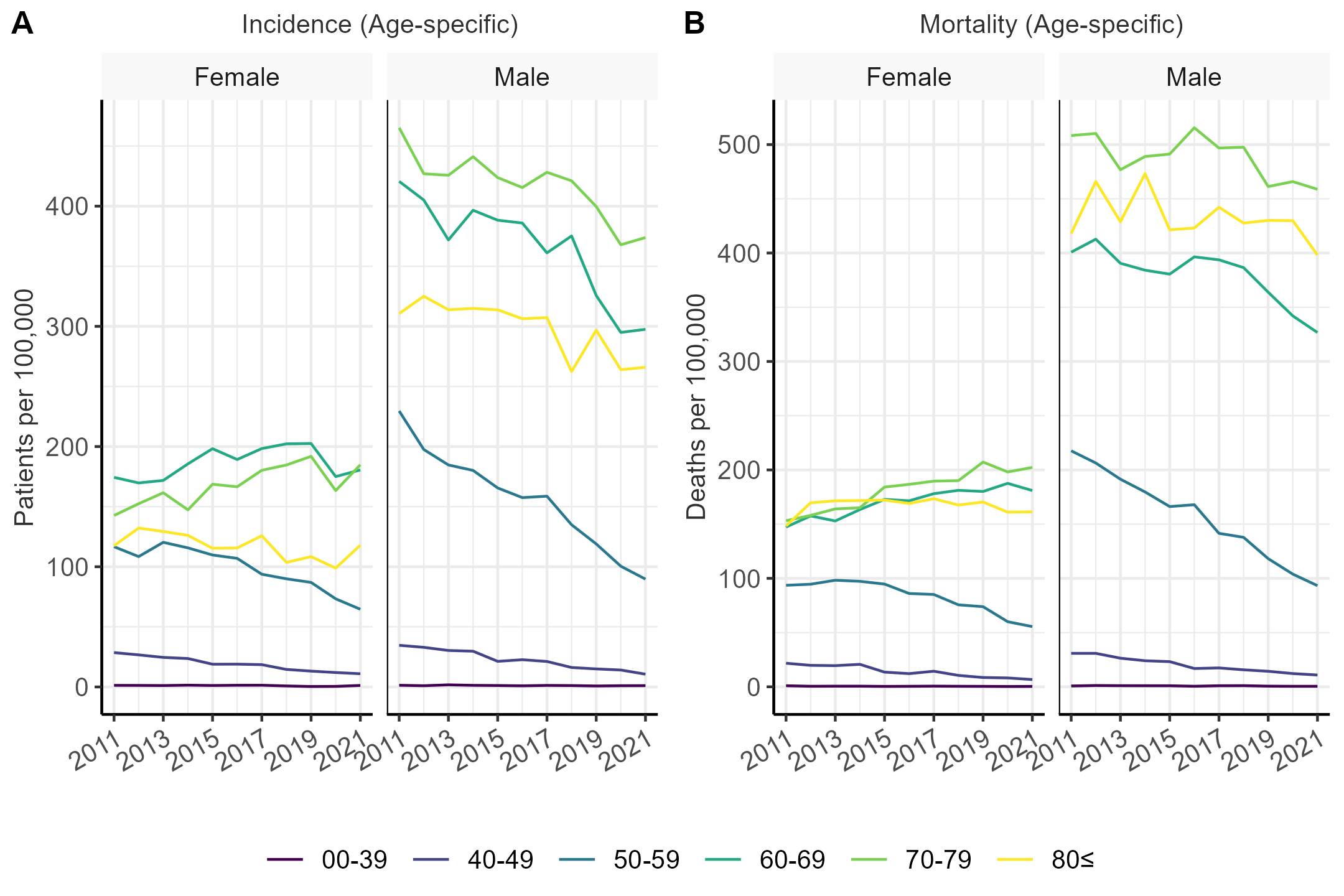

Supplement: Supplementary file 4 [file DataSheet1.ZIP › figures/fig2.png]

## Female

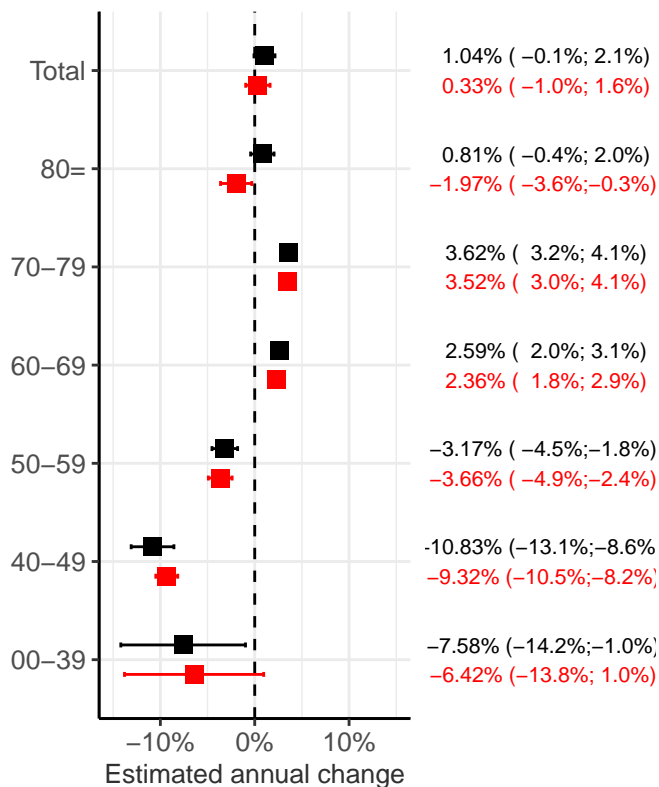

## Male

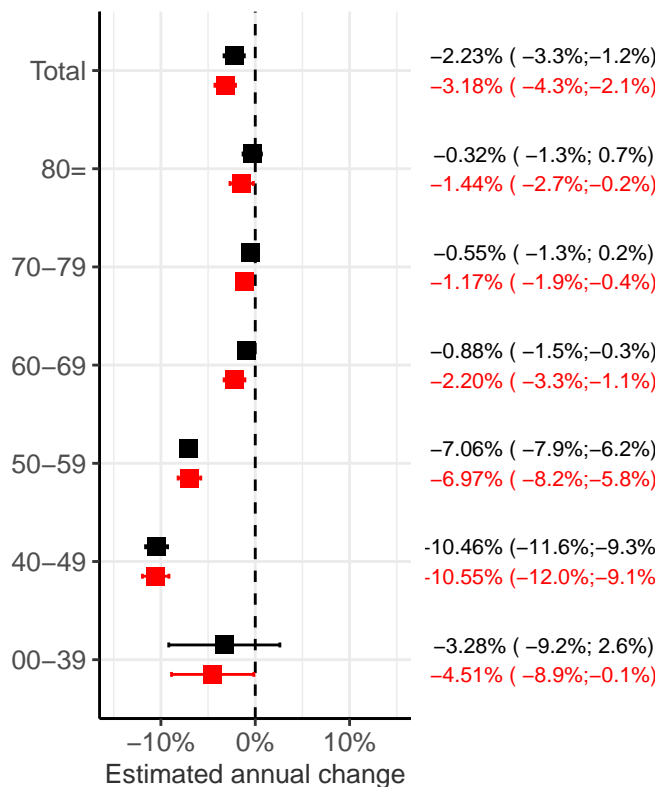

■ Incidence ■ Mortality

Supplement: Supplementary file 4 [file DataSheet1.ZIP › figures/fig3.pdf]

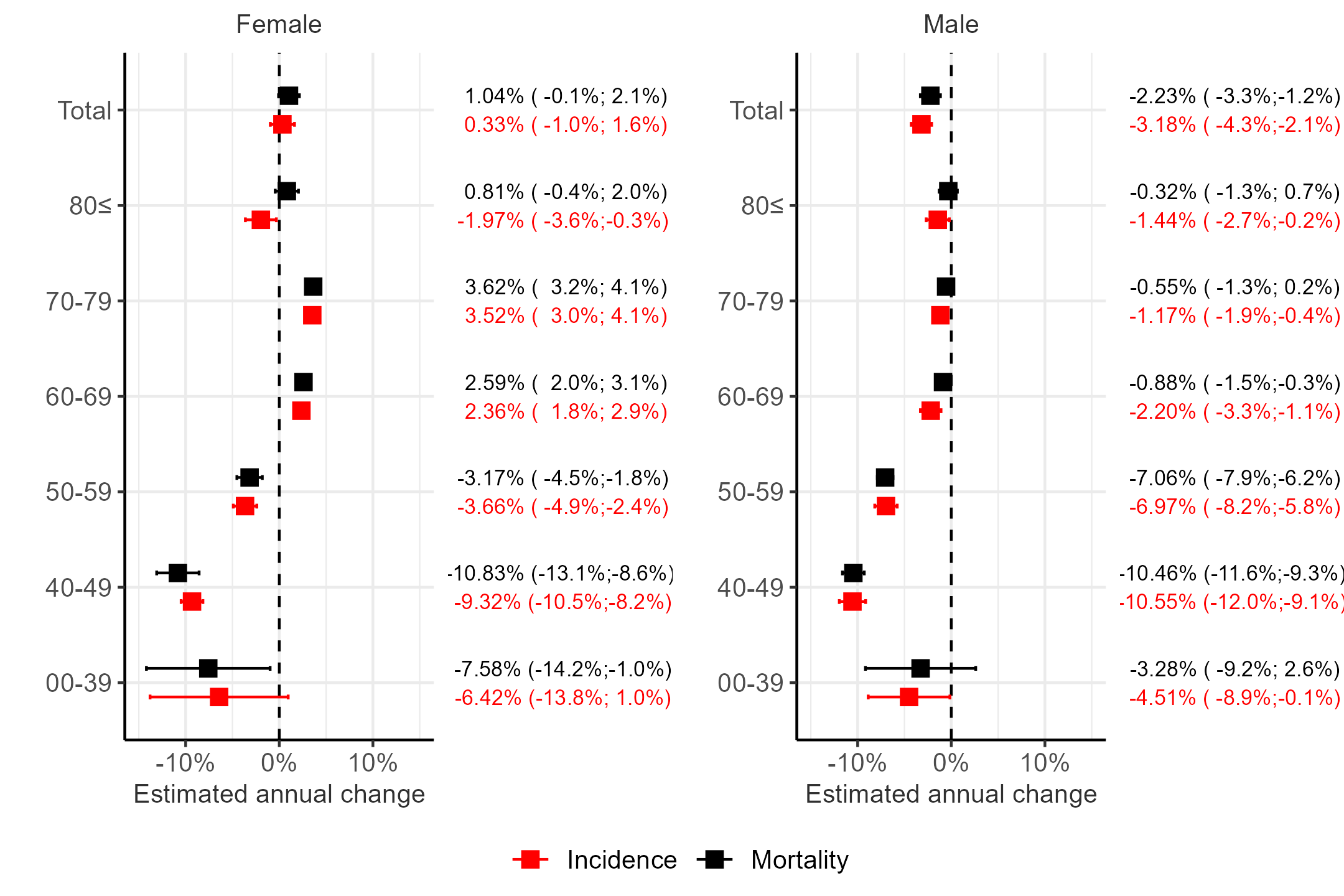

Supplement: Supplementary file 4 [file DataSheet1.ZIP › figures/fig3.png]

Female

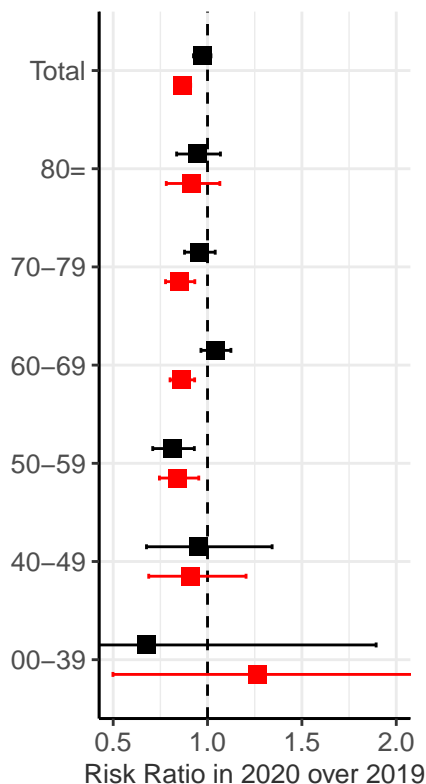

Male

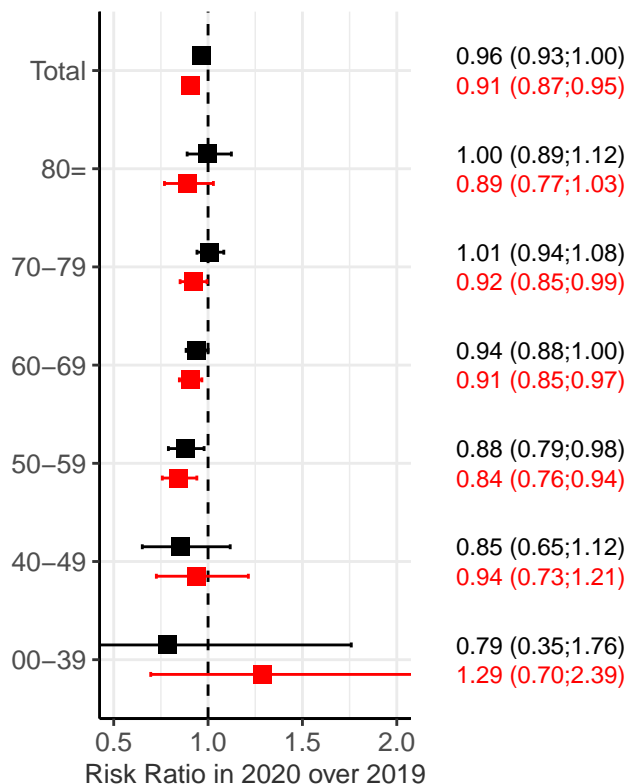

■ Incidence ■ Mortality

Supplement: Supplementary file 4 [file DataSheet1.ZIP › figures/fig4.pdf]

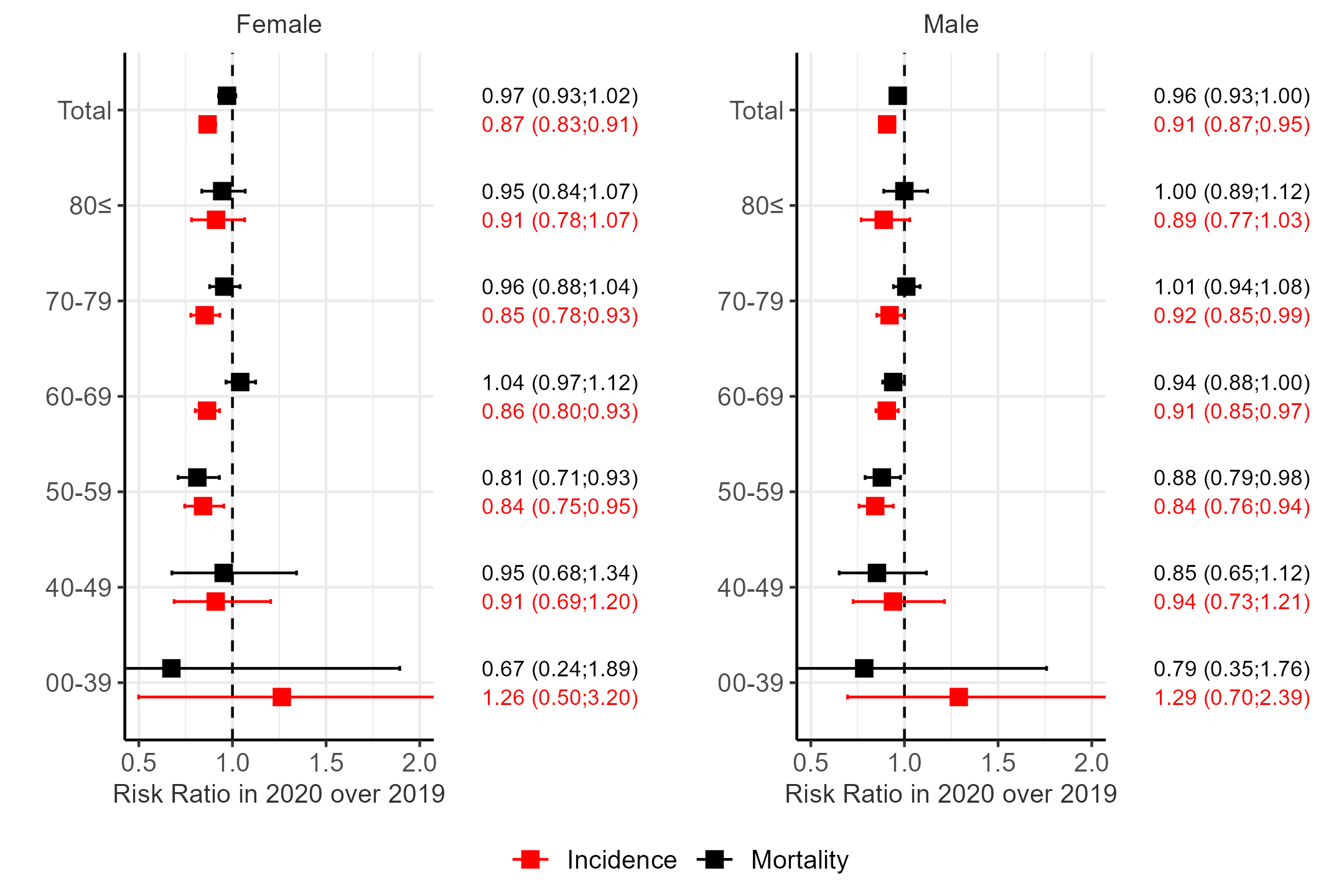

Supplement: Supplementary file 4 [file DataSheet1.ZIP › figures/fig4.png]

Female

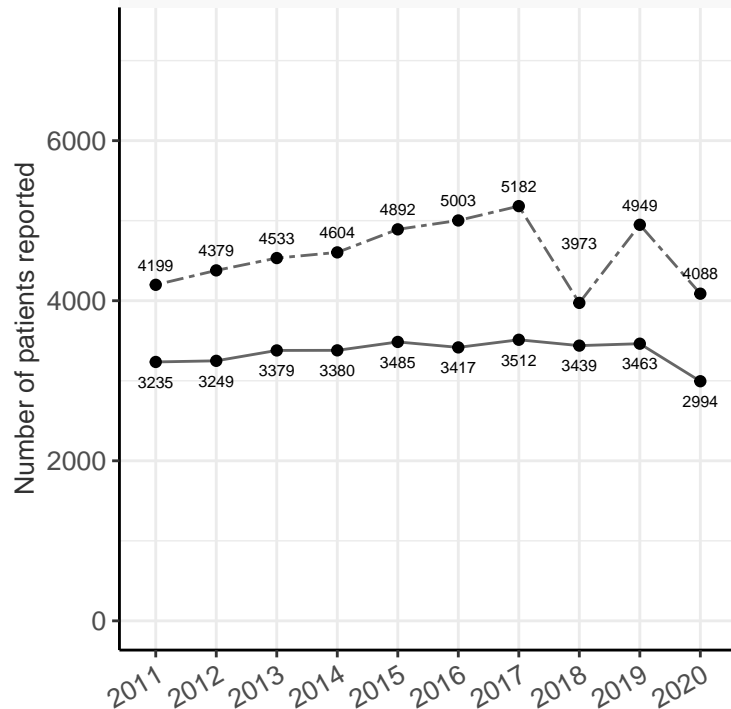

Male

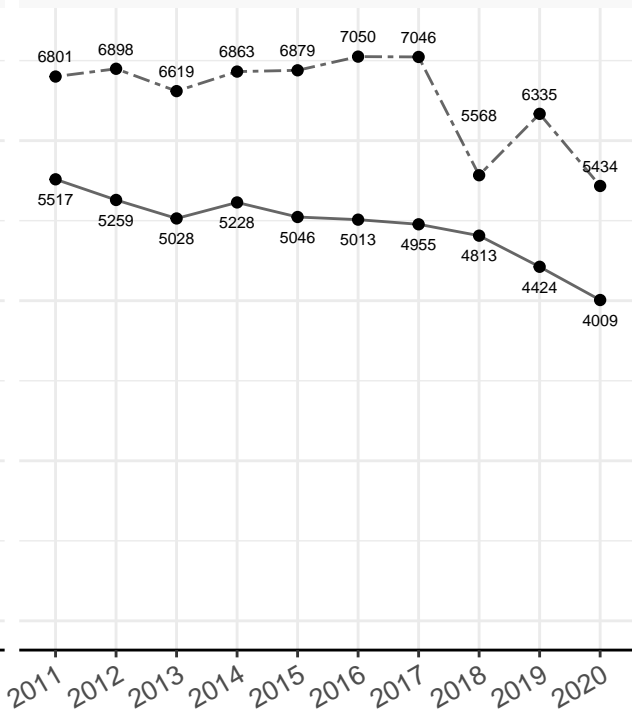

— Optimized    - - - HNCR

Supplement: Supplementary file 4 [file DataSheet1.ZIP › figures/sfig1.pdf]

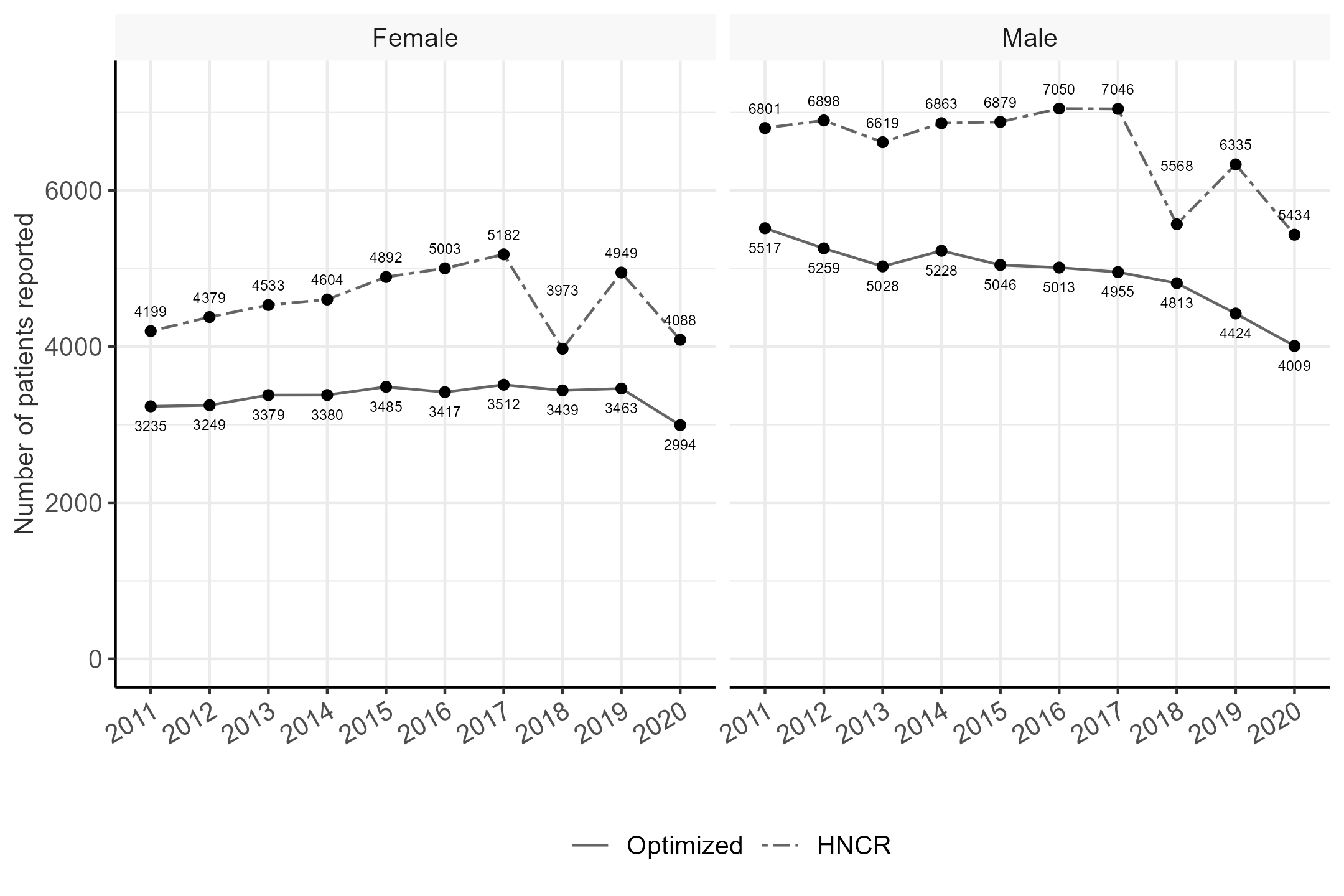

Supplement: Supplementary file 4 [file DataSheet1.ZIP › figures/sfig1.png]

Female

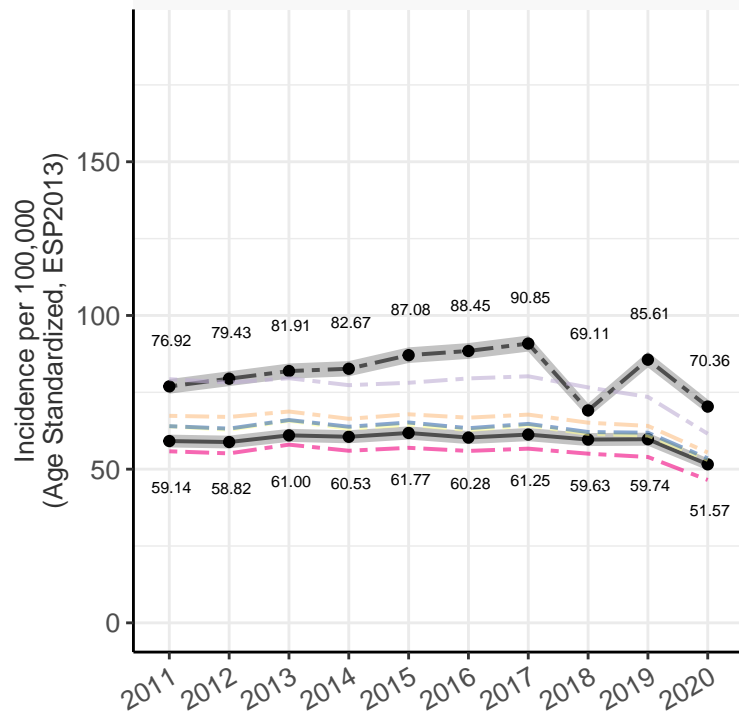

Male

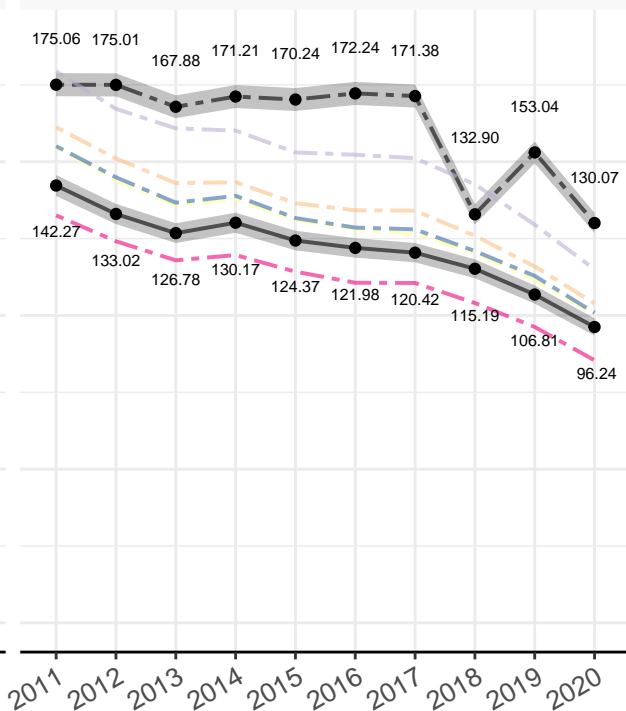

Optimized
  HNCR
  1.1
  1.2A
  1.2B
  1.2C
  1.3

Supplement: Supplementary file 4 [file DataSheet1.ZIP › figures/sfig2.pdf]

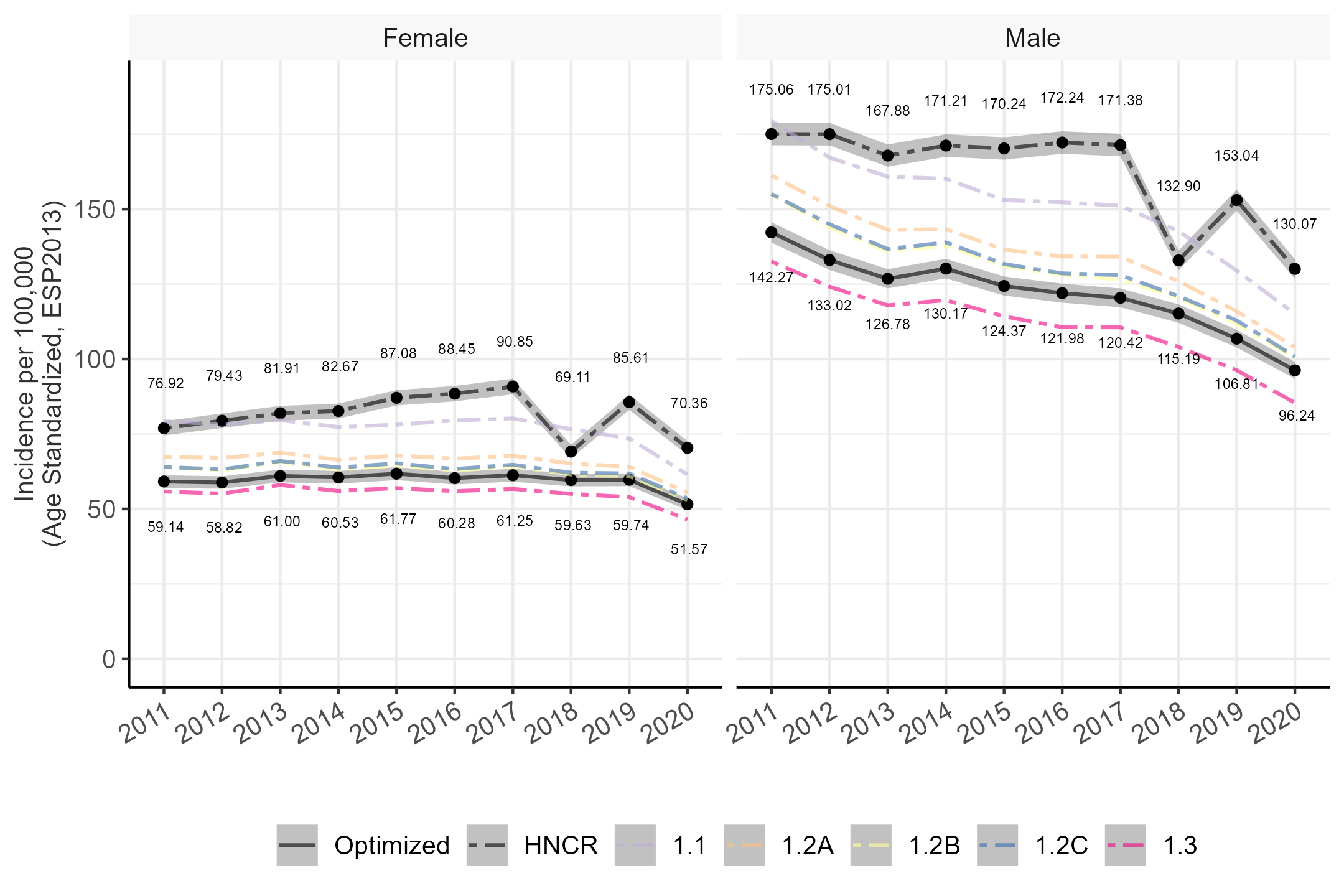

Supplement: Supplementary file 4 [file DataSheet1.ZIP › figures/sfig2.png]

Female

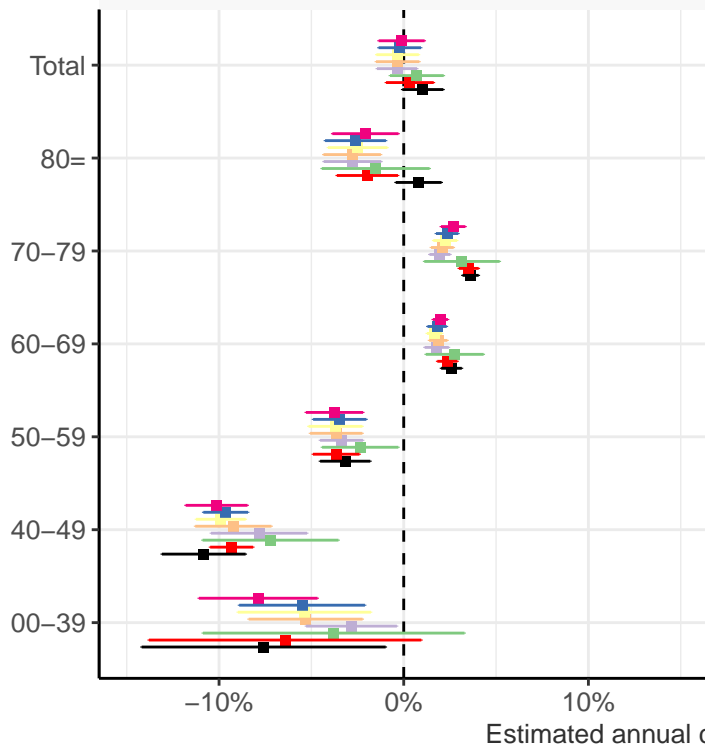

Male

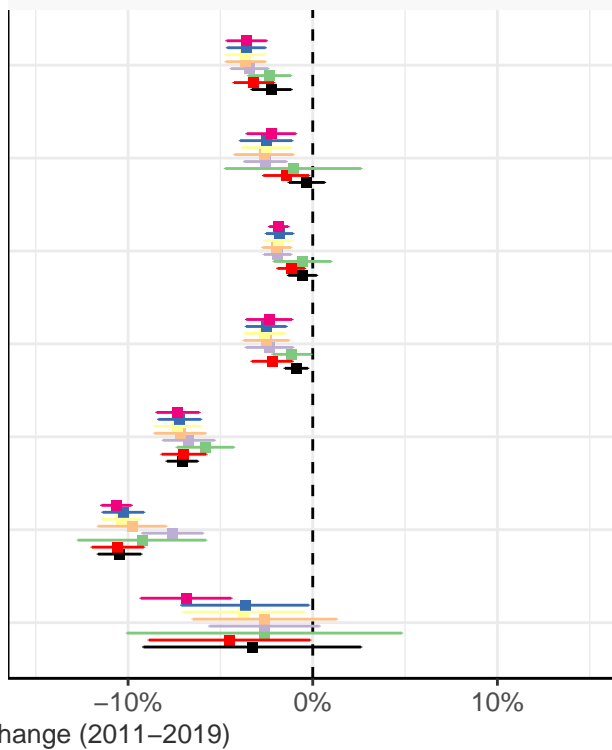

— Mortality — Incidence — HNCR — 1.1 — 1.2A — 1.2B — 1.2C — 1.3

Supplement: Supplementary file 4 [file DataSheet1.ZIP › figures/sfig3.pdf]

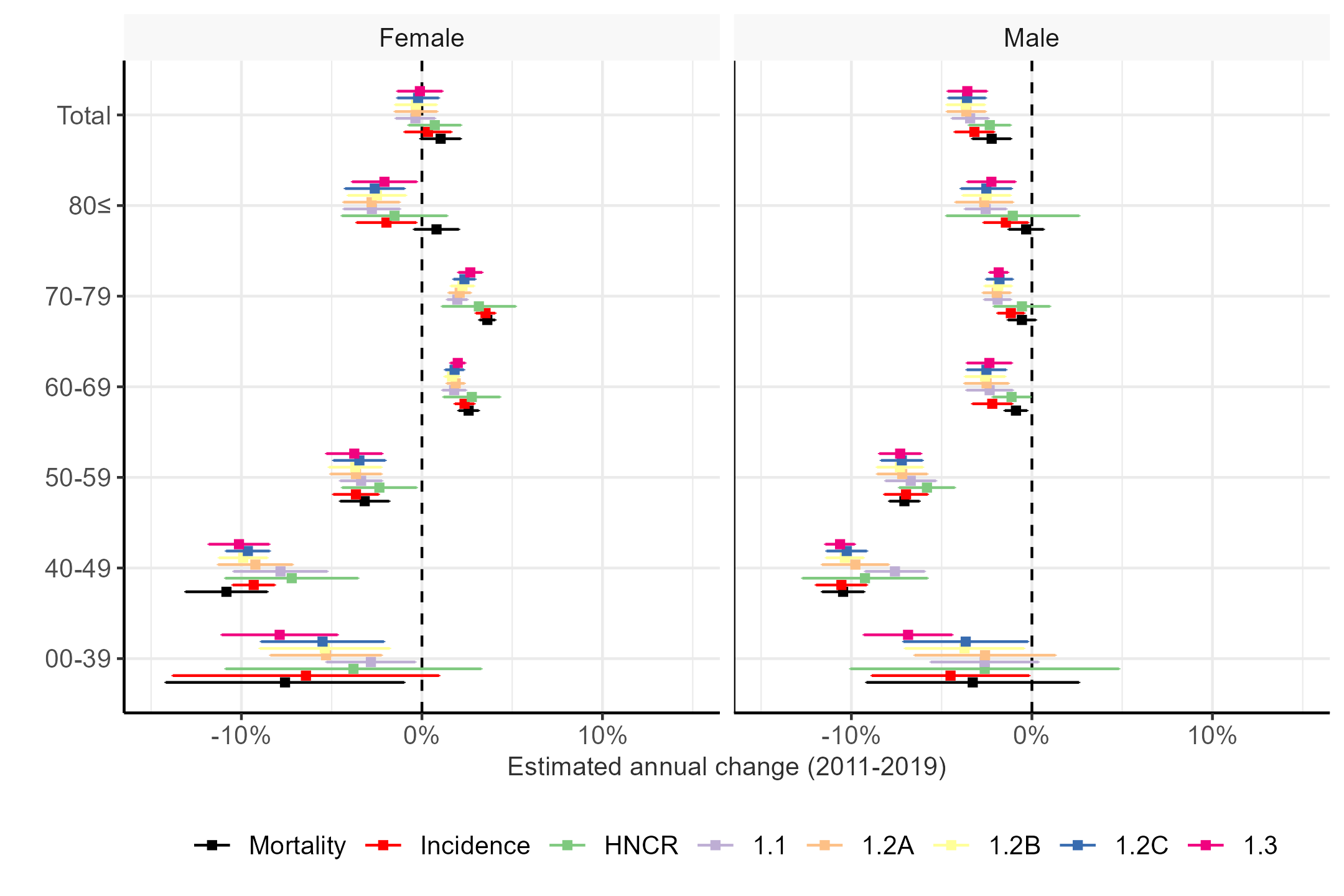

Supplement: Supplementary file 4 [file DataSheet1.ZIP › figures/sfig3.png]

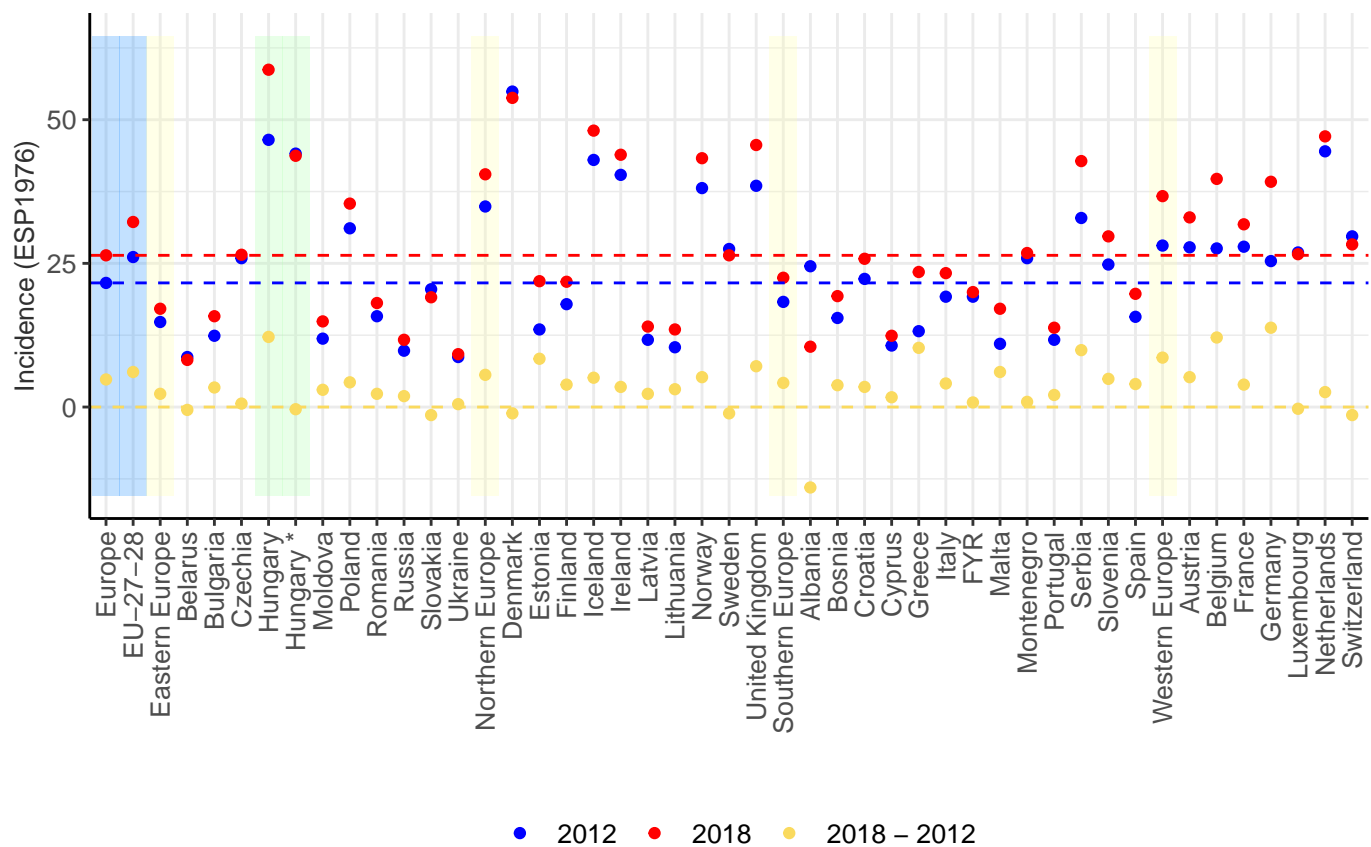

Supplement: Supplementary file 4 [file DataSheet1.ZIP › figures/sfig4.pdf]

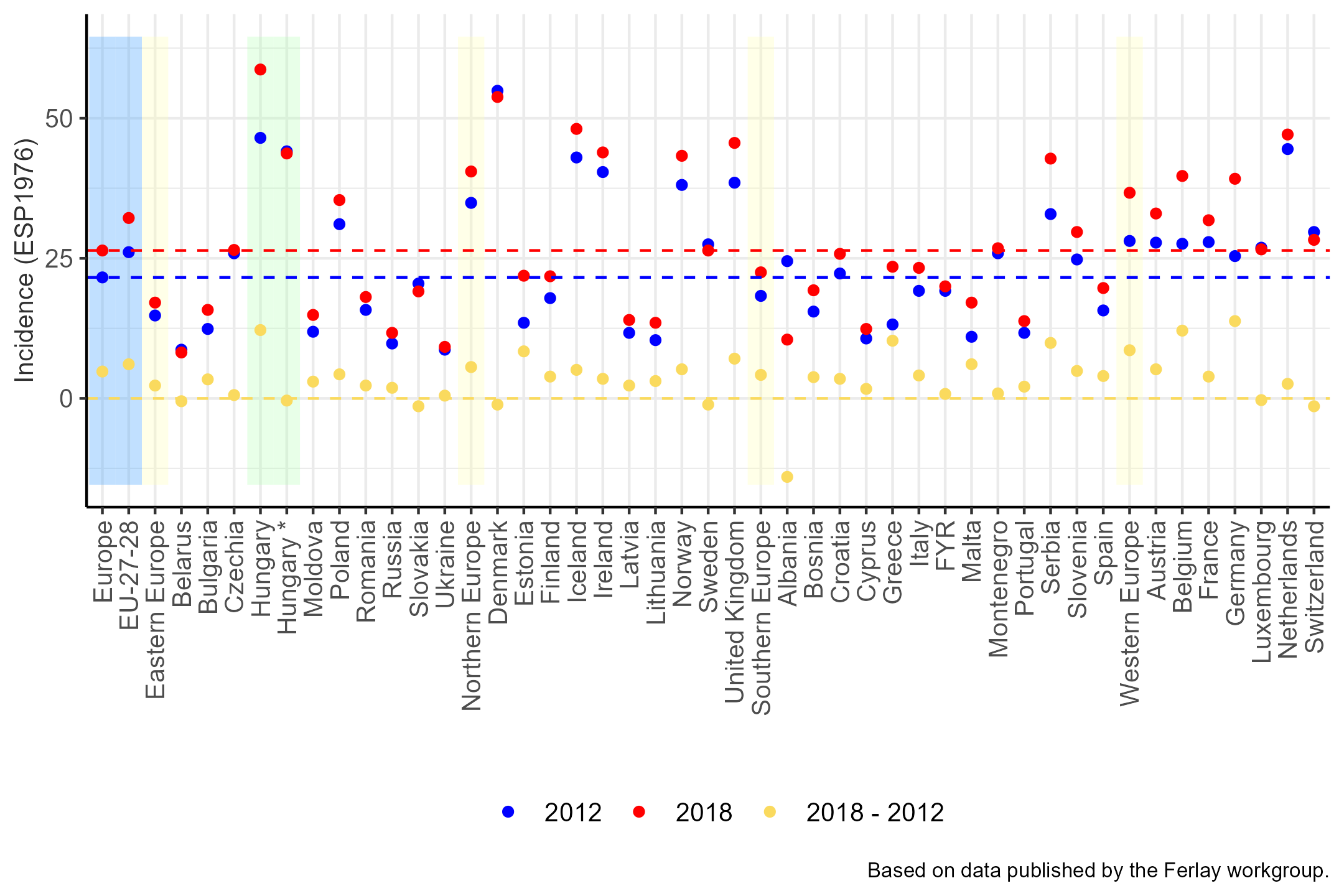

Supplement: Supplementary file 4 [file DataSheet1.ZIP › figures/sfig4.png]

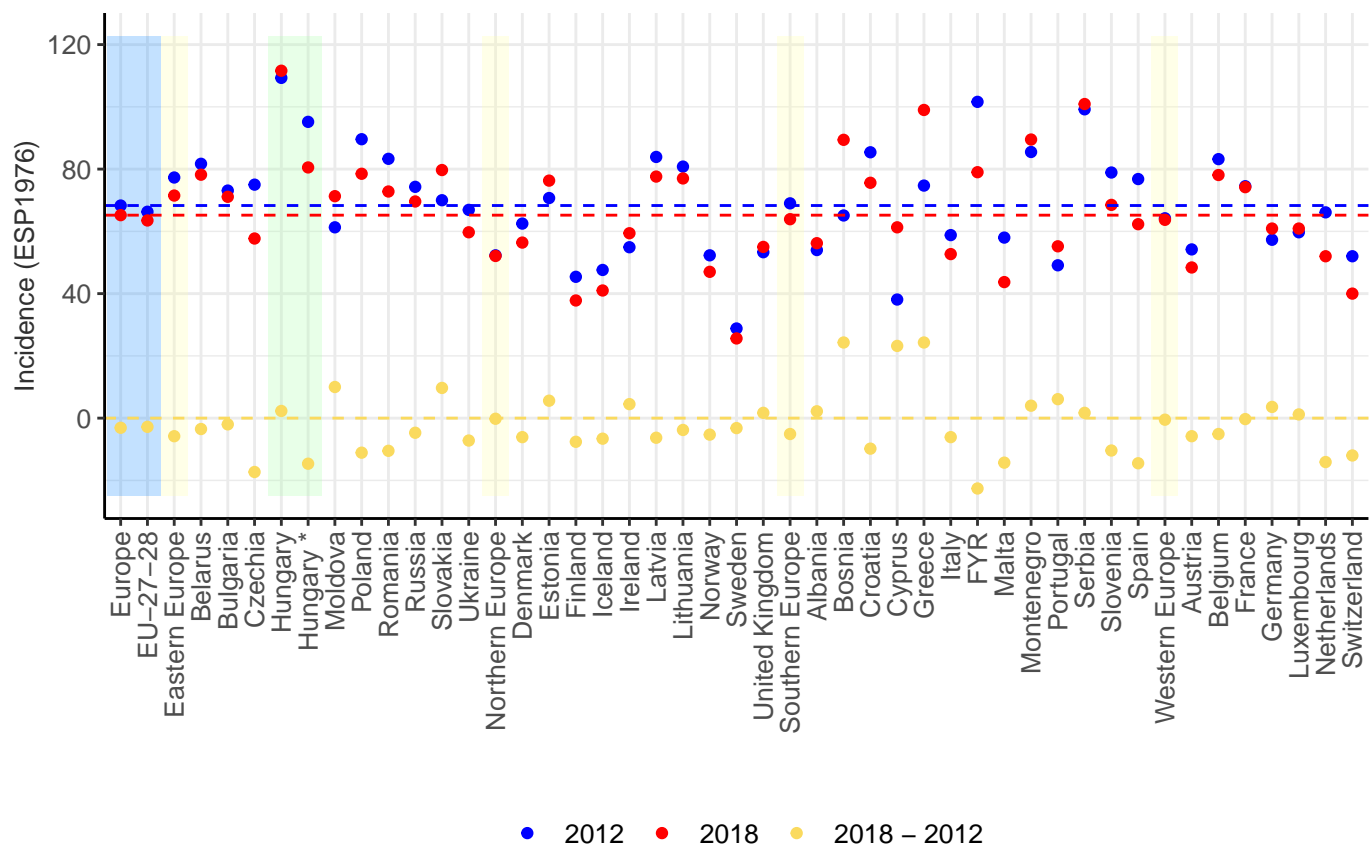

Supplement: Supplementary file 4 [file DataSheet1.ZIP › figures/sfig5.pdf]

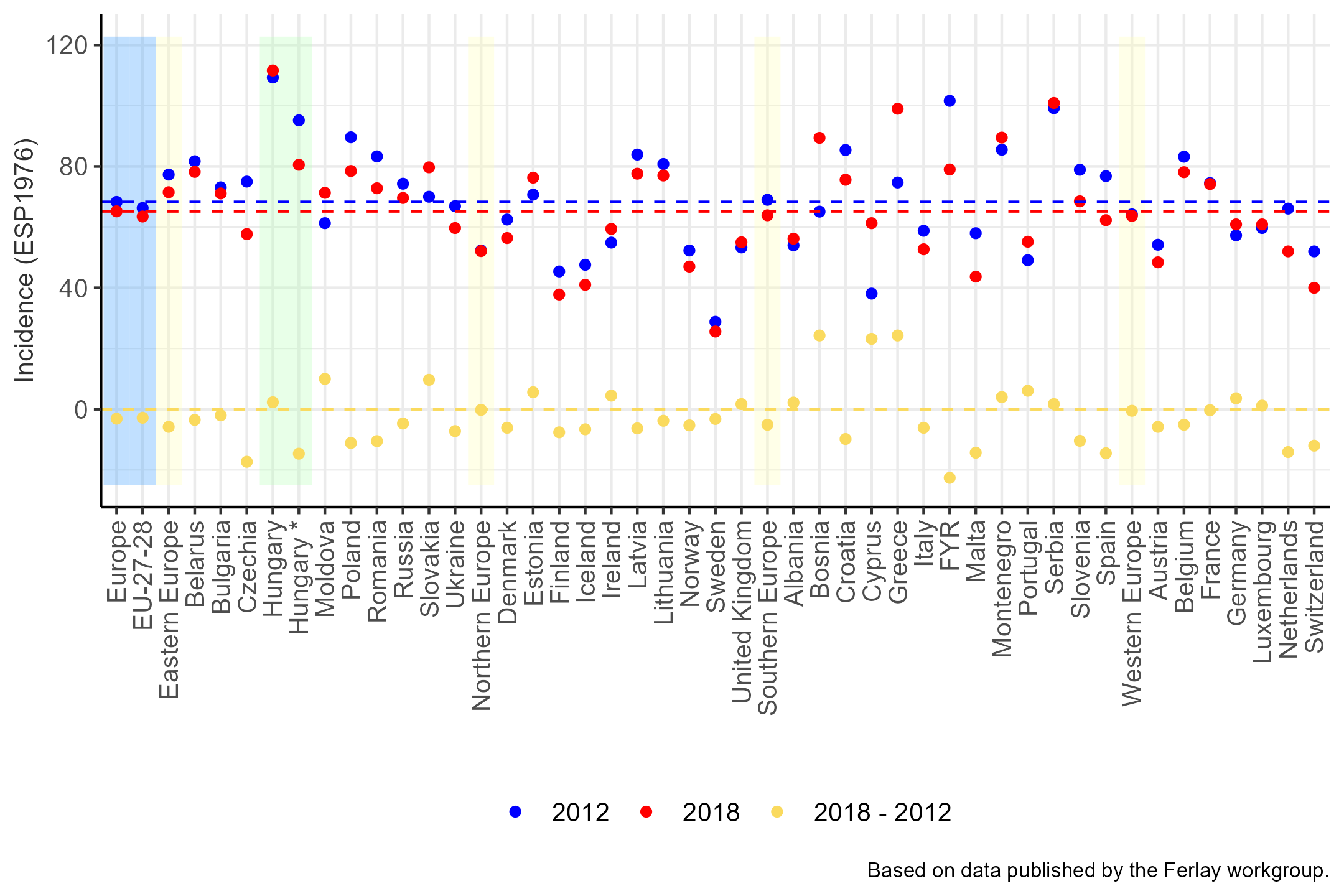

Supplement: Supplementary file 4 [file DataSheet1.ZIP › figures/sfig5.png]

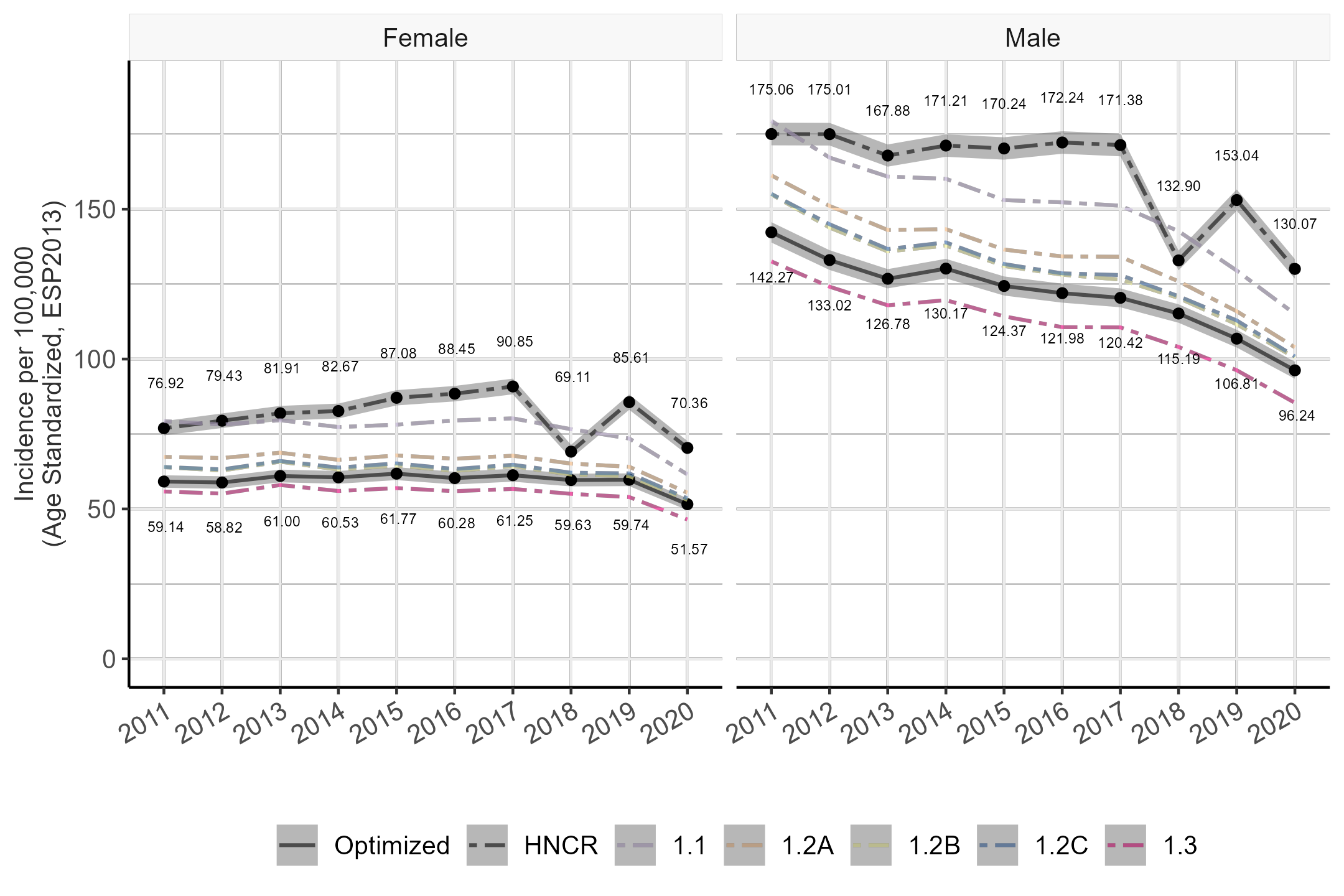

Supplement: Supplementary file 5 [file Image2.TIFF]

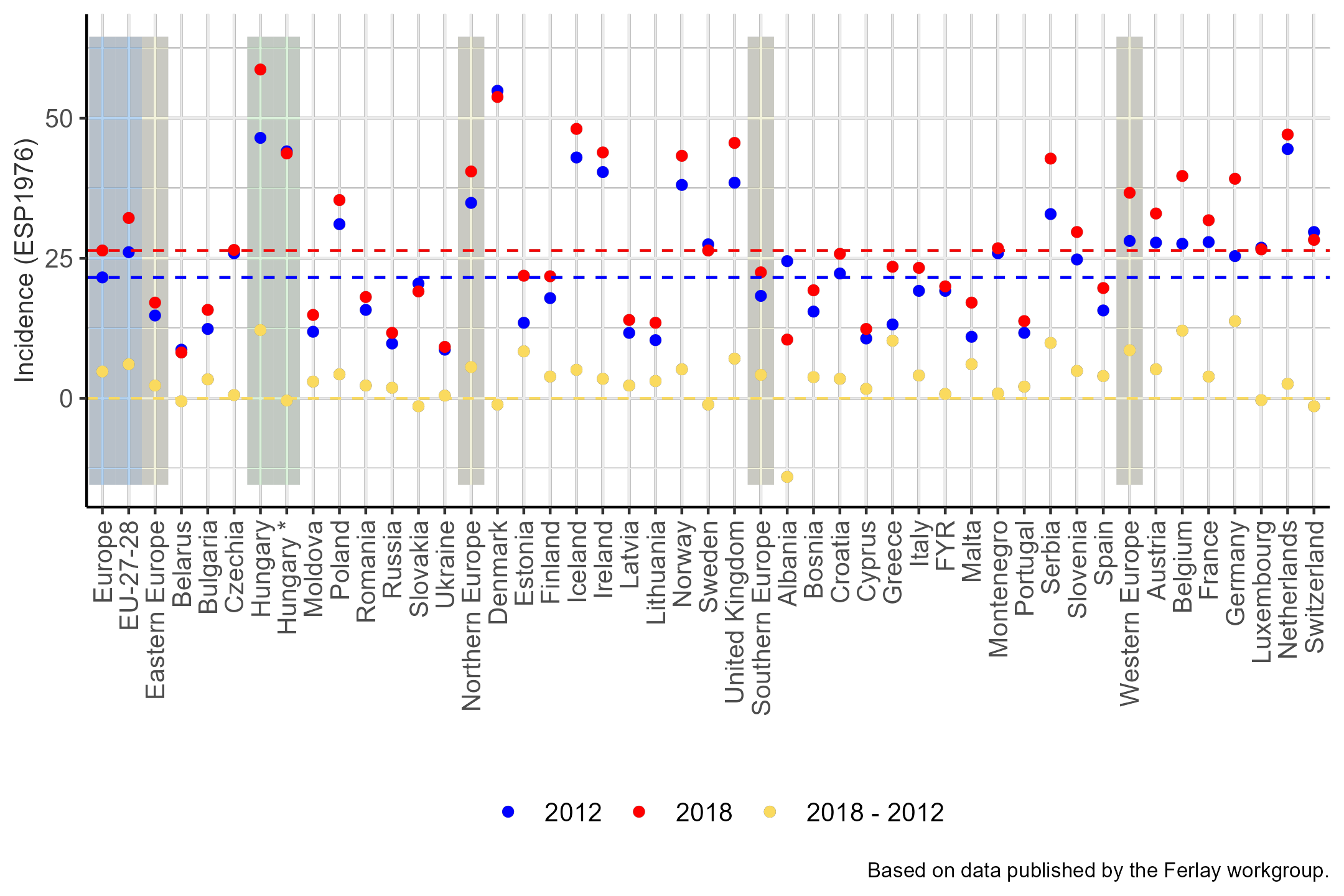

Supplement: Supplementary file 6 [file Image4.TIFF]
